# Development of Molecular Marker Linked with Bacterial Fruit Blotch Resistance in Melon (*Cucumis melo* L.)

**DOI:** 10.3390/genes11020220

**Published:** 2020-02-19

**Authors:** Md. Rafiqul Islam, Mohammad Rashed Hossain, Denison Michael Immanuel Jesse, Hee-Jeong Jung, Hoy-Taek Kim, Jong-In Park, Ill-Sup Nou

**Affiliations:** 1 Department of Horticulture, Sunchon National University, Sunchon, Jeonnam 57922, Korea; rafiqul@sau.edu.bd (M.R.I.); m.r.hossain@bau.edu.bd (M.R.H.); michaelijesse@gmail.com (D.M.I.J.); gml79wjd@sunchon.ac.kr (H.-J.J.); htkim@sunchon.ac.kr (H.-T.K.); jipark@sunchon.ac.kr (J.-I.P.); 2 Department of Biotechnology, Sher-e-Bangla Agricultural University, Dhaka 1207, Bangladesh; 3 Department of Genetics and Plant Breeding, Bangladesh Agricultural University, Mymensing 2202, Bangladesh

**Keywords:** BFB, InDel, inheritance, length polymorphism, melon, NBS-LRR

## Abstract

Bacterial fruit blotch (BFB) causes losses in melon marketable yield. However, until now, there has been no information about the genetic loci responsible for resistance to the disease or their pattern of inheritance. We determined the inheritance pattern of BFB resistance from a segregating population of 491 F_2_ individuals raised by crossing BFB-resistant (PI 353814) and susceptible (PI 614596) parental accessions. All F_1_ plants were resistant to *Acidovorax citrulli* strain KACC18782, and F_2_ plants segregated with a 3:1 ratio for resistant and susceptible phenotypes, respectively, in a seedling bioassay experiment, indicating that BFB resistance is controlled by a monogenic dominant gene. In an investigation of 57 putative disease-resistance related genes across the melon genome, only the MELO3C022157 gene (encoding TIR-NBS-LRR domain), showing polymorphism between resistant and susceptible parents, revealed as a good candidate for further investigation. Cloning, sequencing and quantitative RT-PCR expression of the polymorphic gene MELO3C022157 located on chromosome 9 revealed multiple insertion/deletions (InDels) and single nucleotide polymorphisms (SNPs), of which the SNP A^2035^T in the second exon of the gene caused loss of the LRR domain and truncated protein in the susceptible accession. The InDel marker MB157-2, based on the large (504 bp) insertion in the first intron of the susceptible accession, was able to distinguish resistant and susceptible accessions among 491 F_2_ and 22 landraces/inbred accessions with 98.17% and 100% detection accuracy, respectively. This novel PCR-based, co-dominant InDel marker represents a practical tool for marker-assisted breeding aimed at developing BFB-resistant melon accessions.

## 1. Introduction

Melon (*Cucumis melo* L., 2*n* = 2× = 24) belonging to the Cucurbitaceae family produce delicious, sweet, fruits rich in vitamins, minerals and health-promoting antioxidants [1]. They are an economically important agricultural crop cultivated and consumed all around the world, with an average production of more than 29 million tons per year throughout the past decade [2]. Melons are predominantly cultivated in temperate and tropical countries with all the major producing regions in the world (China, Turkey, Iran, India, Kazakhstan, USA, Egypt, Spain, Guatemala, Italy, etc.) (http://www.fao.org/faostat/en/#rankings/countries_by_commodity). Production is greatly hampered by several pathogens creating a barrier to attaining higher yields of this important fruit crop.

Bacterial fruit blotch (BFB), caused by the aerobic, Gram-negative, rod-shaped and seed-borne bacterium *Acidovorax citrulli* (formerly *Acidovorax avenae* subsp. *citrulli*) [3], is a destructive disease responsible for significant economic losses of melon crops worldwide. It is very difficult to estimate the exact economic losses caused by BFB; however, it can cause 80% to 100% loss in melon production under favorable environmental situations, especially during rainy seasons and highly fluctuating temperature regimes [4,5,6]. The disease also causes significant economic losses in other cultivated cucurbits, such as citron melon, prickly paddy melon, pumpkin, cucumber, squash, several types of gourds and watermelon, and has been reported all over the world, including the United States, Nicaragua, Costa Rica, Brazil, Australia, Turkey, Japan, Korea, Thailand, Taiwan and China [7,8,9,10,11,12,13]. In South Korea, BFB in melon was first reported in 1990, with several outbreaks observed in the last decade [14]. BFB was initially thought to be a severe threat mainly to watermelon. However, recent worldwide increases in BFB outbreaks on other cucurbits, especially melon, are believed to be due to changes in the population structure and worldwide spread of the pathogen [10,12,15,16,17,18].

Melon seedlings and fruits are highly susceptible to *A. citrulli.* Typical symptoms include water-soaked lesions on cotyledons, leaves and fruit that are often small and irregular and progress throughout the leaf and rind of the fruit, leading to collapse and death of the plant [19]. The disease constitutes a severe threat, particularly because it affects marketable yield, i.e., the fruits, as well as the plants [20].

As of yet, chemical and other cultural practices have not been fully effective in managing BFB. Melon growers rely on chemical treatments to control BFB, even though these show only partial success [15,21,22,23,24]. Host resistance represents the most cost-effective and environmentally friendly approach for BFB management [20]. There has been little research to screen sources of resistance to BFB in melons, although a screen of 332 *Cucumis* sp. accessions identified only four *C. melo* and one *Cucumis ficifolius* accessions resistant to BFB [25]. Furthermore, Bahar, et al. [26] reported four genotypes, 6401, BLB-B, EAD-B and ADIR, that were resistant in seedling transmission assays. The genetic control of BFB resistance in melons has not been extensively investigated and no molecular markers linked to resistance to BFB that can be used for breeding BFB-resistant melon cultivars via marker-assisted selection.

Plant resistance to disease is mainly manifested by *R* (resistance)-genes [27,28]. *R*-gene-mediated recognition of pathogen effectors activates a series of defense signaling cascades and stimulates pathogenesis-related gene expression, creating global, durable and broad-spectrum systemic acquired resistance in plants [29]. *R*-genes have been reported in several plant species, including *Arabidopsis*, cucumber and rice [30,31,32], and nucleotide binding site (NBS)-encoding genes throughout the genome have recently been identified in melons [33]. The largest class of known *R*-genes comprises those containing NBS and leucine-rich repeat domains (NBS-LRR proteins). The NBS-LRR (NL)-type *R*-genes are fundamental for plant responses to various pathogens, including bacteria, fungi, viruses and nematodes [30,32]. TIR-NBS-LRR (TNL) proteins containing a Toll-like domain, and CC-NBS-LRR (CNL) proteins characterized by a coiled-coil domain in the *N*-terminal portion represent a subset of NBS-LRR proteins that often function as *R*-genes in plant genomes [34].

Several NBS-LRR-type genes are linked to disease resistance in melons. For example, TNL genes are found in the vicinity of genetic loci controlling resistance to *Fusarium* wilt and papaya ringspot virus [35]. No such quantitative trait locus (QTL) or *R*-gene has been linked with BFB so far. The lack of confirmed molecular markers associated with BFB resistance hampers the transfer of resistance loci to commercial melon cultivars. In this study, we determined the mode of inheritance of BFB resistance and developed a molecular marker linked to BFB resistance in melons by identifying polymorphism in NBS-coding genes across the genome.

## 2. Materials and Methods

### 2.1. Plant Materials and Population Development

An F_2_ segregating population consisting of 491 plants was developed using melon genotypes PI 614596 and PI 353814. The parental accessions PI 614596 and PI 353814, reported to be susceptible and resistant, respectively, to BFB [18,25], were obtained from the U.S. National Plant Germplasm System (https://npgsweb.ars-grin.gov/gringlobal/search.aspx), U.S. Department of Agriculture, USA. The resistance and susceptibility of these accessions were confirmed using a controlled inoculated bioassay [18]. In addition, 22 melon accessions (Supplementary Table S1) were used for validation of the marker. All plants were raised in 32-cell trays containing artificial soil mix in a controlled plant growth chamber with 25 ± 2 °C, 16-h-day lengths and 440 μmol/m^2^/s light intensity at bench level for 3 weeks and then transferred to a greenhouse of the Horticulture Laboratory of Sunchon National University, South Korea.

### 2.2. Bacterial Culture and Inoculum Preparation

*A. citrulli* strain KACC18782 was obtained from the Korean Agricultural Culture Collection (KACC), South Korea. Bacteria were grown in King’s B (KB) media [3] supplemented with 100 μg mL^−1^ ampicillin for 36–48 h at 28 °C until formation of bacterial colonies. For all inoculations, a bacterial suspension was prepared by inundating culture plates with 5 mL of sterile, double-distilled water and gently scraping the surface of the KB media using a sterile, L-shaped rubber spreader to an optical density of 1.0 at 600 nm, as measured using a NanoDrop ND-1000 spectrophotometer (NanoDrop Technologies, Wilmington, DE, USA). Bacterial suspension was diluted to a final concentration of ~1 × 10^6^ colony forming units (mL^−1^).

### 2.3. Inoculation, Sample Preparation and Assessment of BFB Resistance

Plants with 3–5 true leaves (4–5 weeks old) were inoculated by spraying with a suspension of *A. citrulli* KACC18782 using a hand-pump spray bottle until runoff in a greenhouse with a temperature range of 20–25 °C. Inoculated plants were covered with a plastic cover to maintain high relative humidity (96%). Plants were re-inoculated 3 days after the first inoculation to ensure that no plants had escaped inoculation and to eliminate false positives. Samples BFB-resistant and susceptible plants were collected from the fourth and fifth true leaves stage of the plants at the time points of 12 h, 1 day, 3 days and 6 days after inoculation and from control plants at the same time points. The samples were immediately frozen in liquid nitrogen (N_2_). The disease severity of three individual leaves from each inoculated plant was scored 12 days after inoculation using a scale of 1 to 6 (Supplementary Figure S1) for ≥10%, 11–20%, 21–50%, 51–75%, 76–90% and >90% diseased/infected area per leaf, respectively [18]. Disease ratings were scored as per the methods described by Robin et al. [36] and Hassan et al. [37] with little modification. The percentage infected area (PIA) was measured as the ratio of infected area to total leaf area, multiplied by 100. Individuals with a PIA of ≤20 and >21 were considered as resistant and susceptible, respectively [18,37]. Supplementary Table S2 and the resulting ratio was tested for goodness-of-fit using χ^2^ analysis.

### 2.4. Selection of Putative R-Genes and Primer Design

A total of 57 NBS, LRR, TIR and CC domains containing putative disease resistance-related genes covering all 12 chromosomes of melons with particular emphasis on chromosomes that harbor disease and insect resistance-related loci were used for detection of length polymorphism between contrastingly resistant melon genotypes (Table 1). DNA coding sequences were retrieved from the melon (DHL92) genome v3.6.1 from the Cucurbit Genomics database (http://cucurbitgenomics.org/organism/3). 

Moreover, for the quantitative RT-PCR expression analysis of polymorphic (MELO3C022157) gene-specific primers were designed F: CGAATTTGAGTGCTGTTCCA and R: CGAAGTTCTACTGTTGGGCG on the fourth exon. Primers (Table 1), one pair for shorter genes and multiple pairs for longer genes, were designed to cover the entire length of the chromosomes using the Primer3Plus (http://www.bioinformatics.nl/cgi-bin/primer3plus/primer3plus.cgi) web tool. Primer sequences were checked for potential self-dimer and hairpin formation using an oligo calculator (http://biotools.nubic.northwestern.edu/OligoCalc.html).

### 2.5. Extraction of Genomic DNA and Detection of DNA Polymorphism

Genomic DNA (gDNA) was extracted from fresh 3-week-old leaf samples of the two parental accessions (20 from each parent), 20 F_1_ plants, 491 F_2_ plants and 22 melon accessions using a DNeasy Plant Mini Kit (QIAGEN, Hilden, Germany) according to the manufacturer’s instructions. The concentration of extracted gDNA was determined using a NanoDrop Spectrophotometer ND-100 (NanoDrop Technologies, Wilmington, DE, USA) and stored in a refrigerator at −20 °C for further use. PCR was performed in 20 μL reaction mixtures containing 1 μL gDNA (152 ng/μL); 1.0 µl (10 pmol) of each forward and reverse primer; 8 μL Prime *Taq* premix (2×) (GENETBIO Inc., Gwangmyaong, Korea) and 9 μL ultra-pure H_2_O. PCR was carried out in a thermo-cycler with initial denaturation at 95 °C for 5 min, followed by 35 cycles of denaturation at 95 °C for 30 s, annealing at 58 °C for 30 s and elongation at 72 °C for 45 s and final elongation at 72 °C for 7 min. Electrophoresis of amplified PCR products was performed in 1.2% agarose gel stained with HiQ blue mango (20,000×) (bioD, Gwangmyaong, Korea) and visualized using ultraviolet light in a ENDURO™ GDS gel documentation system (New York, NY, USA).

### 2.6. Cloning and Sequencing of the Polymorphic Gene

The polymorphic gene MELO3C022157 was PCR-amplified from resistant (PI 353814) and susceptible (PI 614596) accession using Phusion^®^ High-Fidelity DNA Polymerase (New England Biolabs, EVRY Cedex, France). Amplified DNA fragments were purified using a Promega DNA Purification kit (Promega, Madison, WI, USA). Cloning was performed using a TOPO-TA cloning kit (Invitrogen, Carlsbad, CA, USA) following the manufacturer’s instructions. The universal primers M13F and M13RpUC were used to sequence cloned amplicons using an ABI3730XL sequencer (Macrogen Co., Seoul, Korea). Each forward and reverse sequence of resistant and susceptible melon accession was repeated three times to remove all uncertainties. Gene sequences of the resistant and susceptible accessions were aligned using CLUSTALW software (https://www.genome.jp/tools-bin/clustalw) to detect sequence variation.

### 2.7. Total RNA Extraction and cDNA Synthesis

Control and infected melon leaves were crushed to a powder in liquid nitrogen (N_2_), and 100 mg of each sample was subjected to total RNA extraction using an RNeasy Mini kit (Qiagen, Valencia, CA, USA) following the manufacturer’s instructions. First-strand cDNA was synthesized from total RNA with a SuperScript III First-Strand Synthesis System kit (Invitrogen, Gaithersburg, MD, USA).

### 2.8. Quantitative RT-PCR (qRT-PCR) Expression of Polymorphic Gene MELO3C022157

The expression patterns of the polymorphic-gene were analyzed by qRT-PCR in a LightCycler^®^ instrument (Roche, Mannheim, Germany) following the manufacturer’s instructions. The reactions were performed in a 10-µL volume consisting of 5 µL of 2x qPCRBIO SyGreen Mix Lo-ROX (PCR Biosystems, London, UK); 5 pmol of primers and cDNA templates diluted to the appropriate concentrations. The PCR conditions were as follows: 5 min at 95 °C, followed by 3-step amplifications at 95 °C for 15 s, 56 °C for 15 s and 72 °C for 20 s, for 45 cycles. The mean expression level of relevant genes was calculated by the 2^−ΔΔ Ct^ method [38], where *Actin* was used as an internal control [39].

### 2.9. Statistical Analysis

To determine significant changes in gene expression levels among the different time points for each treatment, a chi-square (χ^2^) test for goodness-of-fit was performed to determine deviations of observed data from expected segregation ratios using Minitab18 statistical software (State College, PA, USA). A value of *p* < 0.05 was considered statistically significant. Turkey’s pairwise comparison test was conducted for mean separation.

## 3. Results

### 3.1. Dominant Inheritance of BFB Resistance in Melon

**Figure 1:**
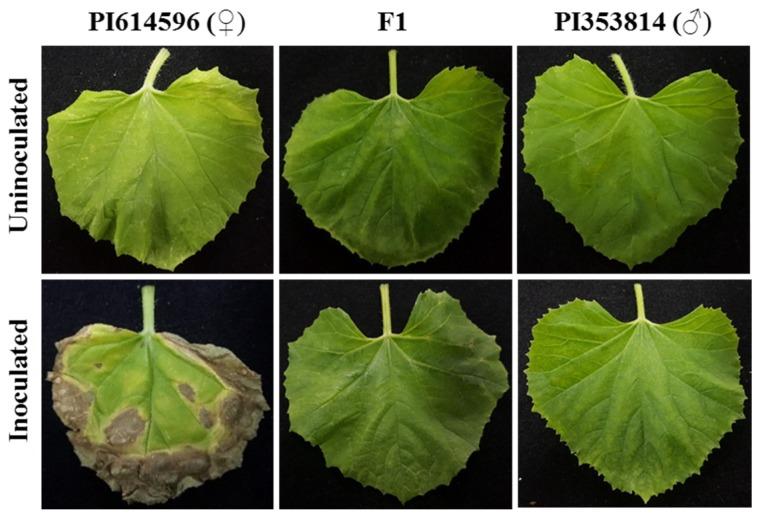
Bacterial fruit blotch phenotypes on leaves of the two parental melon accessions, PI 353814 (resistant) and PI 614596 (susceptible), and their F_1_ hybrid (resistant) 12 days after inoculation with *A. citrulli* strain KACC18782, as compared to uninoculated controls. All leaves were detached just before the photographs were taken.

To determine the inheritance pattern of BFB resistance in melon, we crossed a BFB-resistant (PI 353814) and a BFB-susceptible (PI 614596) parental accession for making F_1_. The resistant parental accessions PI 353814 had less than 20% BFB-infected leaf area, while more than 70% of the leaves of susceptible parental accessions PI 614596 were infected by *A. citrulli* (KACC18782) (Figure 1). Symptoms of the leaves of F_1_ hybrids resembled those of the resistant parent, indicating the dominant nature of the inheritance of this trait in melons. We next evaluated the inheritance of BFB resistance in the F_2_ population consisting of 491 plants.

Bioassay results indicated that 360 F_2_ plants were resistant, and 131 were susceptible. A chi-square (χ^2^) test revealed that BFB resistance segregated with a 3:1 ratio (resistant: susceptible), indicating monogenic dominant control of the trait (Table 2 and Supplementary Figures S2 and S3).

### 3.2. Microsynteny Analysis of 57 R-Genes of Melon Compared with Watermelon and Cucumber

**Figure 2:**
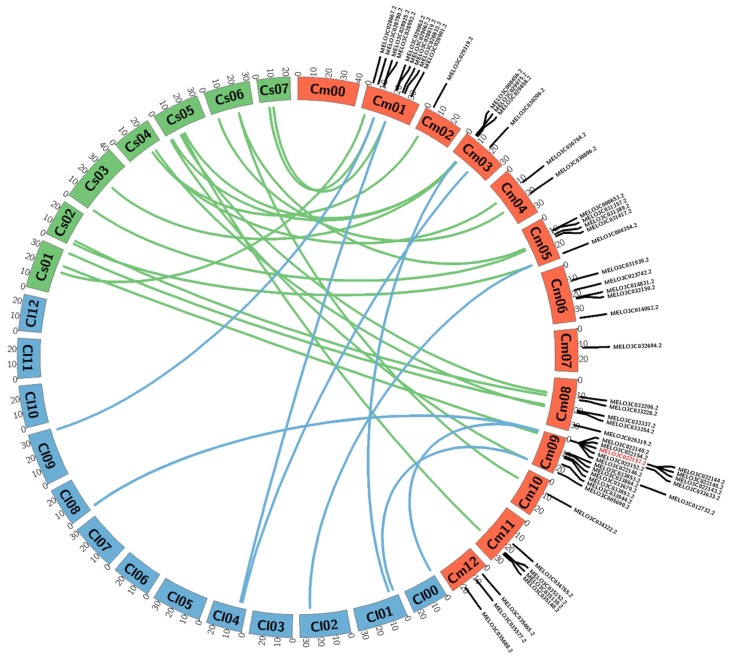
Microsynteny analysis of melon *R*-genes compared with watermelon and cucumber. Brown orange, blue and green indicate melon, watermelon and cucumber chromosomes, respectively. Red marking indicates genes showing polymorphism between the resistant (PI 353814) and susceptible (PI 614596) parents of the melon. Microsynteny analysis of genes on the melon chromosomes were drawn using the web-based tool Circos (http://circos.ca/software/download/) circos-0.69-9.tgz.

We performed comparative analysis to identify the homologous *R*-genes among melon, compared with watermelon and cucumber. Some *R*-genes from melons share homologous relationships with those of watermelon and cucumber. However, only four chromosome genes of melons (chr.01, chr.03, chr.05 and chr.09) share homologous relationships with those of watermelon, while remaining chromosome genes are not homologous. Moreover, genes of melon on chr.06, chr.07 and chr.12 lack homologues in watermelon, whereas other chromosome genes are homologous (Figure 2).

### 3.3. Identification of Length Polymorphisms in Putative R-genes

It is essential to identify BFB-resistant genotypes to develop BFB-resistant melon cultivars. Since no definitive *R*-gene is known to be associated with BFB resistance in melon, we identified polymorphisms in putative *R*-genes with NBS, LRR, TIR and CC domains throughout the genome based on a functional annotation of sequences in the Cucurbit Genomics database (http://cucurbitgenomics.org/search/genome/3).

We selected 57 NBS-encoding genes covering all chromosomes of melon, with a maximum 17 and 9 genes on chromosomes 9 and 1, respectively (Table 1). Among these 57 genes, 44 were NBS-LRR (NL) type, while 17 and 2 were TIR-NBS-LRR (TNL) and TIR-NBS (TN) types, respectively. Only one gene each was selected from the NBS and CC-NBS-LRR (CNL) types. We designed gene-specific primers (one set for shorter genes and multiple sets for longer genes) covering the entire length of these genes for PCR amplification and detection of length polymorphisms between resistant and susceptible accessions. Among these genes, conspicuous length polymorphism was detected only for the disease resistance-related TNL-type gene MELO3C022157 (Supplementary Figure S4).

### 3.4. Cloning and Sequencing of the Polymorphic Gene

**Figure 3:**
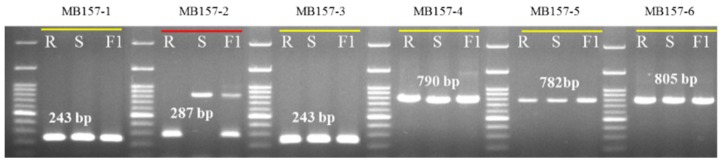
Banding patterns of six pairs of primers designed against the gene MELO3C022157. Polymorphic primer MB157-2 is marked with red underline. R—resistant parent PI 353814, S—susceptible parent PI 614596 and F1—their F_1_ hybrid. Primer details are given in Table 3.

**Figure 4:**
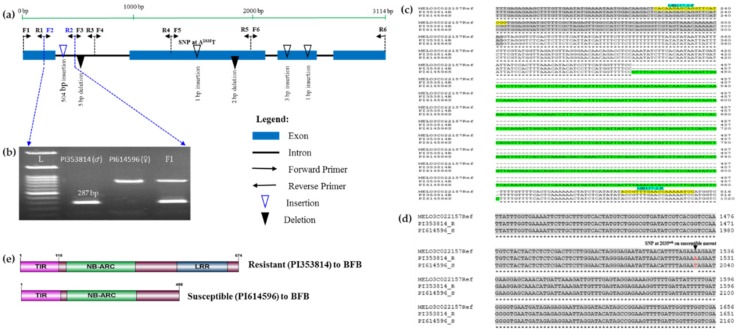
Polymorphism in the TIR-NBS-LRR gene MELO3022157 linked with bacterial fruit blotch (BFB) resistance in melon. (**a**) Idiogram of gene MELO3022157 showing the positions of the six sets of primers used (Table 3) and of insertion/deletion (InDel) and SNP polymorphisms between the resistant (PI 353814) and susceptible (PI 614596) parental accessions. (**b**) Polymorphic PCR amplicons generated by the MB157-2 primer pair in resistant and susceptible parents and their F_1_ progeny. (**c**) Sequence alignment showing a long insertion (green highlighted region) in the susceptible parent. Grey shaded regions indicate exons and yellow highlighted segments indicate MB157-2-F and MB157-2-R primer sequences with primer names marked by blue highlights. (**d**) SNP at the 2035th bp causing a frameshift mutation marker is indicated in red (Supplementary Figure S6b). (**e**) Loss of LRR domain in the susceptible accession. The complete alignment is shown in Supplementary Figure S6b.

We divided the polymorphic gene MELO3C022157 into six consecutive fragments amplified by six sets of primers (Table 3) to narrow down the specific polymorphic region. The polymorphic region was identified in the first intron of the gene ( Figure 3; Figure 4b). We cloned and sequenced the six MELO3C022157 gene fragments from both resistant and susceptible accessions, revealing insertion of a 504 bp fragment in the first intron of the susceptible parent (Figure 4c).

In addition, we detected a 5 bp deletion in the 1st. intron of the resistant accession; a 4 bp deletion in the 2nd. exon of the susceptible accession; three insertions: 1 bp in the 2nd. exon and 3 and 1 bp in the 3rd. exon of the susceptible accession and several single nucleotide polymorphisms (SNPs: “A/G, A/T, A/G and A/T), all ”A” in the resistant parent at positions 1355, 1526, 2454 and 2893 in the 2nd., 2nd., 3rd. and 4th. exons, respectively, and ”G”,”T”, ”G” and ”T” in the susceptible parent at positions 1864, 2035, 2959 and 3398 in the 2nd._,_ 2nd., 3rd. and 4th. exons, respectively, and ”G”,”T” and ”G” in the susceptible parent at positions 1864, 2035 and 2959 in the 2nd._,_ 2nd. and 3rd. exons, respectively (Figure 4a and Supplementary Figure S6b). The SNP ”A/T” (”A” and ”T” in resistant and susceptible parents at positions 1526 and 2035, respectively) generates a premature stop codon in the susceptible parent that produces a truncated protein (Figure 4d, Supplementary Figure S6b,c). In silico domain analysis of the translated protein sequences of the cloned gene of resistant and susceptible accessions using InterProScan (https://www.ebi.ac.uk/interpro/search/sequence-search) revealed that this SNP causes loss of the LRR domain in the susceptible accessions PI 614596, whereas this is present in the resistant accessions PI 353814 (Figure 4e).

### 3.5. qRT-PCR Expression Analysis of Polymorphic Gene MELO3C022157

**Figure 5:**
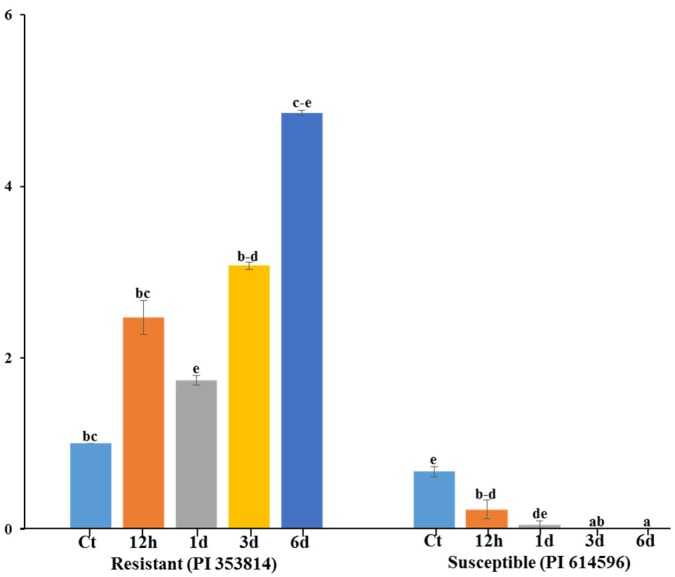
Relative expression levels of putative candidate *R*-gene (MELO3022157) in *A*. *citrulli*-resistant and susceptible melon accession. Error bars represent (± SE) of three individual observations. Different letters above the bars indicate significant differences. Ct—control, h—hour and d—day.

The expression of the polymorphic gene via qRT-PCR gradually increased in the leaf tissue of the resistant (PI 353814), as compared to the susceptible (PI 614596) parent at different time points. The gene was induced within 12 h of infection by *A. citrulli* in both the resistant and susceptible parents. In general, the transcript level in the resistant parent was higher at 12 h, 1 d, 3 d and 6 d after inoculation followed by a decrease in expression (Figure 5) and, after 6d, expression level was very low in the susceptible parent. The gene expression levels revealed a 5-fold peak 6d after inoculation in the resistant parent, whereas transcript levels were lowest in the susceptible parent at this time point.

### 3.6. Development and Validation of InDel Marker Linked to BFB Resistance in Melon

**Figure 6:**
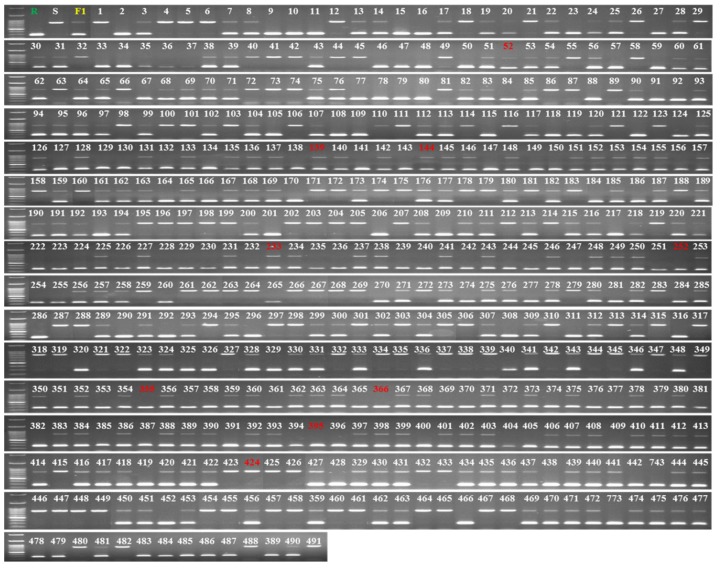
Validation of the InDel marker MB175-2 in 491 plants of an F_2_ population raised from resistant and susceptible parental accessions PI 353814 and PI 614596, respectively. Red numbers indicate accessions with a mismatch between phenotypic and genotypic results.

Among the polymorphisms, we targeted the long insertion/deletion (InDel) mutation between resistant and susceptible accessions and designed a set of primers (MB157-2-F/R) around this mutation (Table 3) by producing a 287 bp amplicon for the resistant accessions, a 791 bp amplicon for the susceptible accessions and both bands for the heterozygous F_1_ hybrid (Figure 4b). To validate the efficiency of the marker, we genotyped 491 F_2_ individuals generated from the resistant and susceptible accessions. The genotypes of 482 individuals matched those from the bioassay results, indicating a detection accuracy of about 98.17% (Figure 6 and Supplementary Table S2). We then used the marker to genotype 22 landraces/inbred melon accessions collected from various sources (Supplementary Table S1), of which we detected two accessions, PI 140471 and PI 420145, as resistant (Figure S5b). A bioassay test of these 22 landraces/inbred accessions indicated that these two accessions were indeed resistant to BFB (Figure S5a). These results suggest that the InDel MB157-2 marker can be effectively used for detecting resistant and susceptible melon genotypes using a PCR-based assay.

## 4. Discussion

Developing BFB-resistant melon accessions is essential, since chemical control measures and other cultural practices are only partially effective, increase the cost of production, are not sustainable and are hazardous to the environment [40]. Even though BFB causes extensive damage, there has been no genetic information about any causal loci or genes controlling the resistants or their pattern of inheritance. Here, we determined the inheritance of BFB disease in melon and developed an InDel marker for distinguishing resistant and susceptible melon genotypes through a PCR assay.

Field evaluation after inoculation with *A. citrulli* strain KACC18782 indicated that seedlings of all F_1_ plants from the cross PI 614596 × PI 353814 were resistant, demonstrating the completely dominant nature of the trait in melon (Figure 1). Similar evaluations of 491 F_2_ individuals showed a Mendelian segregation phenotypic ratio of 3:1 (resistant:susceptible) and the genotypic ratio of 1:2:1 (homozygous (resistant):heterozygous (resistant):homozygous (susceptible)), indicating monogenic control of resistance to the disease (Table 2 and Supplementary Figures S2 and S3).

We then focused on developing molecular markers to detect resistant vs. susceptible genotypes by a simple PCR assay. However, since no QTL or functional gene has been reported for BFB resistance in melon, we examined putative *R*-genes genome-wide.

Functional analysis has revealed that plant disease resistance genes (*R*-genes) mostly encode proteins containing NBS, LRR, TIR, CC and RLK domains [27,28,41], which play roles in defense against various phyto-pathogens, such as bacteria, viruses and fungi [34,42]. For example, NBS-LRR genes control resistance to *Fusarium oxysporum* races 0 and 2, papaya ringspot virus [43] and powdery mildew [44]; a CNL gene controls *Aphis gossypii*-mediated virus resistance [45] and a TNL gene controls resistance against gummy stem blight disease [37] in melon. The NBS domain acts as a molecular switch of the plant defense mechanism, LRR domains are responsible for pathogen recognition and TIR and kinase domains play roles in defense signaling [46,47,48]. Such *R*-genes have been identified throughout the genomes of various crop species. such as rice, *Arabidopsis*, potato, soybean, maize, rapeseed and cabbage [49,50,51,52,53], including different cucurbits, such as cucumber, bottle gourd, luffa, watermelon and squash [30,32,54]. Several studies have identified NBS-encoding *R*-genes genome-wide in melon [33,55,56].

We investigated length polymorphism in 57 putative disease resistance genes encoding proteins containing NBS, LRR, TIR and CC domains and covering all 12 chromosomes of melon, with particular emphasis on chromosomes and regions harboring disease and insect resistance-related loci. Conspicuous length polymorphism between resistant and susceptible melon accessions was only observed for the TIR-NBS-LRR gene MELO3C022157 (Table 1 and Supplementary Figure S4). Besides, the presence of homologues of this *R*-gene in watermelon and cucumber (Figure 3) indicates that this gene is likely to play a similar role in melon [35,37]. After cloning and sequencing of the entire polymorphic gene (resistant and susceptible parent) revealed a long insertion (504 bp) in the first intron in the susceptible parent and several other small InDels and SNPs between the resistant and susceptible parents (Figure 4c and Supplementary Figure S6b). Of these, the SNP A^2035^T in the second exon caused loss of the LRR domain, resulting in a truncated protein in the susceptible accession (Figure 4e and Supplementary Figure S6c). So, the polymorphic gene belongs to the LRR domain; qRT-PCR transcription level is higher at 6d after inoculation in resistants, whilst loss of LRR and transcription level is little at 6d in the susceptible (Figure 4e and Figure 5), indicating that 6d after inoculation in the susceptible parent fails to develop a hypersensitive response, i.e., causing BFB disease, suggesting that the LRR domain may be responsible for the susceptible reaction of the mutant accession. LRR domains are involved in resistance against a yellow strain of cucumber mosaic virus (CMV[Y]) in *Arabidopsis* [57,58], which strongly support our results. LRR domains also implicate *LepR3* loci mediate resistance against blackleg in *Brassica napus* [59] and against *Fusarium* wilt (*Fom2*) in melon [57,58]. Since LRR domains provide specificity in pathogen recognition [48,60], the NBS-LRR class of plant innate immune receptors uses its LRR domain to accomplish many other roles [61].

In developing a molecular marker, we targeted the 504 bp InDel region in the first intron of MELO3C022157 and designed primer pair MB157-2-F/R, which identified resistant and susceptible accessions among 491 F_2_ individuals with 98.17% detection accuracy and those representing 22 landraces/inbred accessions with 100% detection accuracy (Table 3, Supplementary Tables S1 and S2 and Figure 6 and Supplementary Figure S5b). Introns play diverse roles in processes such as transcription coupling [62], gene expression regulation, formation of noncoding RNA [63,64] and exon shuffling and alternative splicing [65]. In addition, intronic polymorphism is a valuable source for developing genetic markers with high interspecies transferability [66,67], since introns are under much lower selective pressure than other functional elements such as exons [68]. Several markers have been developed using intronic polymorphism for a range of traits in different crops, including cabbage, melon, maize and sunflower [37,66,69,70]. Notably, a pair of InDel markers located in the same first intron of MELO3C022157 are reportedly linked to gummy stem blight resistance in melon [37].

## 5. Conclusions

We determined that BFB resistance in melon is controlled by a single dominant gene, allowing the development of the PCR-based co-dominant InDel marker MB157-2 for identifying resistant and susceptible accessions. The co-dominant InDel marker MB157-2 developed in this study will have practical implications for marker-assisted breeding for improving BFB resistance in melons. This is the first report, to our knowledge, of a molecular marker linked to BFB resistance in melons. Work is underway to map the resistant loci using partial genome sequence-based approaches.

## Figures and Tables

**Table 1:** Details of 57 putative disease resistance-related genes and corresponding primers used for detecting length polymorphism between resistant and susceptible melon genotypes.

Sl.	Gene ID	Chr.	Domain	Primer	Forward Primer (5′-3′)	Reverse Primer (5′-3′)	Amplicon Size (bp)
1	MELO3C028667	chr01	NL	MB8667	ATGGGGTGCAATTATGGAGA	TTACTCCATTCTACACCGCG	192
2	MELO3C028780	chr01	NL	MB780	ATGCATTCTAGGAGCAATCA	CTAGGGTAGGCTAGGATTGG	240
3	MELO3C028925	chr01	NL	MB925	GGATTCCAGCGAGGAGATTG	CTACCGTGGATTGCAAACAC	938
4	MELO3C028952	chr01	NL	MB952	GGATTGACATTTCCTCTGCA	CTAGGGATCATGTGGTGGTC	488
5	MELO3C029063	chr01	NL	MB063	ATGGGCCGTGCATTCCAGGA	TCACACTCGTTGTCCTGCGG	243
6	MELO3C029067	chr01	NL	MB067	ATGGCCTTCACGCATCCTTC	TTATTGAGTCGTCTTCGAAG	897
7	MELO3C028819	chr01	NL	MB819	GCGGCCGCGACGAATGAACG	CCCCTTTCGCTTCGTCTTCT	169
8	MELO3C028832	chr01	NL	MB832	GACAACCACTCTCTGAGGAT	AAGAAATCACCAAGCGTTAG	237
9	MELO3C028901	chr01	NL	MB901	ATGAGATATGCGATCAAGTG	GCCATACATCTCCTAGCGAG	259
10	MELO3C029319	chr02	NL	MB319	CATACCCAGTAAGGGACCCA	CCCACACTTGGGACATGACT	696
11	MELO3C029975	chr03	NL	MB975	ATGGCGTCAGAGATAGCGTC	TCTGCTTTTCAATCTGTTCC	666
12	MELO3C029858	chr03	N	MB858	ATGGGTTTCCGAAAGGGATT	CAGAATTTCATCAGGGATCC	186
13	MELO3C008456	chr03	NL	MB456	CCACAAGAATACATCAAGGT	TGGTATCGCATATCTCTTCC	289
14	MELO3C030206	chr03	NL	MB206	GGGTAAGTTGATCTTTCACA	CGCGACTCCAAGCCGACCCG	1560
15	MELO3C030764	chr04	NL	MB764	GGTTCTTGGCTTGCAACTAG	TTAGGTGGATCCGTTGCGCG	535
16	MELO3C030896	chr04	NL	MB896	ATGGCATTGGGAATAGGGGG	TCACACGGTCAACCAGCTTC	279
17	MELO3C000653	chr05	NL	MB653	CTTACGGCTCGGCTCGGCTC	GTCGTCGAACGCCAAAGACC	258
18	MELO3C031357	chr05	NL	MB357	CGGCATAAAAGACGTCGGGA	TCCCATCCTCAGAGAGTGGC	334
19	MELO3C031389	chr05	NL	MB389	ATGCAAGAGCAATTACGGAC	ACTTCAATCCGTTCCAAAGC	442
20	MELO3C031417	chr05	NL	MB417	ATGAATCCCACCTGGCGGCA	TTAGCTTGAACGATATTGCG	348
21	MELO3C004354	chr05	CNL	MB354-1	ATGGCGGGAGCTTTAATTGG	ATCTAAGGTGTCTTTGAGGC	1209
				MB354-2	GCCTCAAAGACACCTTAGAT	TCATGCATATAGCCAATCCA	1256
22	MELO3C031939	chr06	NL	MB939	GCAATCACGGACGAACAAGG	TCATCTACCCAACACCTGAT	197
23	MELO3C023742	chr06	NL	MB742	ATGAGATATGCGAGATCGTG	CTATGCCTGACTCATCTCCT	270
24	MELO3C014831	chr06	NL	MB831	ATGCGGCGGAGGGCAGCTGG	TCATCGGTTCTCTTTCTCCT	270
25	MELO3C032150	chr06	NL	MB150	GGATTGACACTTCCTCTACG	TTACACCTTCGGTCTTTCCA	540
26	MELO3C014062	chr06	TN	MB062-1	ATGGATTCTGATGGGGTCGA	AACCATCTTGTATCTTTGGG	1437
				MB062-2	CCCAAAGATACAAGATGGTT	TCAGATCTTGCTCCGAAGCC	1538
27	MELO3C032694	chr07	NL	MB694	CCAAGCCTACCCCAAAGGGT	CCTCGTATGCCATTCTACAC	194
28	MELO3C033206	chr08	NL	MB206	ATGGCCTTCACGCGTCCTTC	TAGCACTTTCTTCTGATGGG	388
29	MELO3C033228	chr08	NL	MB228	ATGCATGAGCAATCACGAAC	CAAAAAGTTCACACCGCAAG	519
30	MELO3C033337	chr08	NL	MB337	GCAATCATGGACGAACAAGG	TGCTCAATTAGGACTTTAGC	349
31	MELO3C033354	chr08	NL	MB354	ATGAAGAACTTAAAGCTTGA	GGAATGACGTATTTCCAATG	417
32	MELO3C026319	chr08	NL	MB319	TTGGGGCTCGCGACTCAGCT	TCAGCCGACAGCACTACCGT	477
33	MELO3C033633	chr09	NL	MB633	ATGCGCATAATCAAATGCTG	ACGTCATTTTACATTGCGGG	181
34	MELO3C033853	chr09	NL	MB853	GGATTGACACTTCCTTTTCG	TTACATGCGCATCCTCGACG	242
35	MELO3C033864	chr09	NL	MB864	ATGCAGGAGCAATCACGAAC	CTAACGCTGCATGATTTCTT	378
36	MELO3C033679	chr09	NL	MB679	GATAGGCACTTCCTCTGCGA	CTAGCTCACCTCGTTGCTTA	237
37	MELO3C012732	chr09	NL	MB732	CGGCCGCGACGAAAGGACGT	CCTCTTCCGCTTCGTCTTCT	180
38	MELO3C033893	chr09	NL	MB893	GAGATAAACCGTTCGTGTCC	TCATACGTCTTCTAACGAAC	198
39	MELO3C033944	chr09	NL	MB944	ATGCATTCCAGCAATCACGG	CTAGGGATCATGTGGTGGTC	585
40	MELO3C005690	chr09	TN	MB690-1	ATGGACGTTGGAGAAGAAAG	TATATCGGCTTTCGCCTCCA	1522
				MB690-2	TGGAGGCGAAAGCCGATATA	AGTTTCACGAGCAGATATCG	1444
41	MELO3C022157	chr09	TNL	MB157	ATGGAAGCAATTGAGGAATC	TACAATGACCTAGTACTCCC	733
42	MELO3C022154	chr09	TNL	MB154-1	CGAAGATACACGTGGCGGTT	TTCTCCCATTCATCCAACCC	1272
			TNL	MB154-2	GGGTTGGATGAATGGGAGAA	TGGCCTCCTTCTCTTCTTCC	2044
43	MELO3C022152	chr09	TNL	MB152	ATGGCTTCTCCAGCAACAAT	TATATAGTTACCTGATCCCG	1664
44	MELO3C022149	chr09	TNL	MB149	CTCCTTCTCCTCCTTATTCT	TTATATCCTCACGGAGCCAC	1241
45	MELO3C022148	chr09	TNL	MB148-1	GAAGGGGCCATCAAAGAAAT	TTGGGATGGAAATTTGGAGG	1298
			TNL	MB148-2	CCTCCAAATTTCCATCCCAA	ATGTAAAGAGAGAGAGAGAG	998
46	MELO3C022146	chr09	TNL	MB146-1	GAGGCGAAGATACTCGTAAT	AAGTTCTTACTGGGAAACCC	1090
			TNL	MB146-2	GGGTTTCCCAGTAAGAACTT	GCGTGATGGAGTTGAGGGGG	1164
47	MELO3C022145	chr09	TNL	MB145-1	ATGGCTGCAGGTTCCTCATC	CAAAGCTAATGGGAGTCTTC	1329
			TNL	MB145-2	GAAGACTCCCATTAGCTTTG	AACTTGGTGGAAGTTTGTGC	1194
			TNL	MB145-3	GCACAAACTTCCACCAAGTT	CTAGATTTGGCCTAATGTTG	1324
48	MELO3C022144	chr09	TNL	MB144-1	ATGCAGAGTTCATCATCGTC	GACTATAACCAAAACTCTCC	1426
			TNL	MB144-2	GGAGAGTTTTGGTTATAGTC	GGAGAGTTTTGGTTATAGTC	1596
			TNL	MB144-3	GACTATAACCAAAACTCTCC	TCATTGAATTTGAGGCTCCT	1920
49	MELO3C022143	chr09	TNL	MB143-1	ATGGCTTCCTCCACCACCAC	ATTCACCACAGTTATGAGGG	1521
			TNL	MB143-2	CCCTCATAACTGTGGTGAAT	TCAACCCCCATTCTCCCAAG	1523
50	MELO3C034322	chr10	NL	MB322	ATGAGCTTCAGGAATACCAT	CTAACGCTTGGTGATTTCTA	588
51	MELO3C034765	chr11	NL	MB765	CGACAAGTACAAGCAGTTCC	TCAGCTTTCGCATTTGTTCC	165
52	MELO3C035130	chr11	NL	MB130	ATGCATTCCAAGAGCAATCA	GCATCGAAATAACTACTCCC	298
53	MELO3C035140	chr11	NL	MB140	ATGCCTCTTCTCCGCAACCT	ATCCTTCGGGATCACTAGAC	460
54	MELO3C035152	chr11	NL	MB152	ATGCAGGAGAAAGCACGGAC	CTACCCTCTGAGGTAGGTTG	237
55	MELO3C035465	chr12	NL	MB465	ATGATTTCAACTTTTAATAT	CTAAATCCACCGGTGCCTGA	309
56	MELO3C035577	chr12	NL	MB577	ATGGCTCGGATGGTTGATGG	CTTGTACTTCAATCCGTTCC	333
57	MELO3C035688	chr12	NL	MB688	CGTCGGAAGTCGTTATTTCC	CTAGGGATCATGTGGTGGTC	567

Chr—chromosome, NL-NBS-LRR, CNL-CC-NBS-LRR, TN-TIR-NBS and TNL-TIR-NBS-LRR. In primer names, MB denotes melon BFB, and numbers represent the last three digits of the respective genes.

**Table 2:** Inheritance pattern of bacterial fruit blotch (BFB) resistance in melons (*Cucumis melo*).

Parent/Cross	Resistant (PIA ≤ 20)	Susceptible (PIA > 21)	Phenotypic Ratio (R:S)	χ^2^	*P*
P_1_ (PI 353814; Resistant)	20	0			
P_2_ (PI 614596; Susceptible)	0	20			
F_1_ (PI 614596; ♀ × PI 353814; ♂)	20	0			
F_2_	360	131	3:1	2.8	0.244

PIA—percentage infected area, P_1_—resistant parent and P_2_—susceptible parent. In the first three rows ”20” indicates: we get 20 resistant (P_1_), susceptible (P_2_) and resistant (F_1_), respectively (out of 20, in all cases), after conducting a controlled bioassay.

**Table 3:** List of primers designed along the entire length of gene MELO3C022157 to narrow down the position of length polymorphism and to clone the gene.

Primer	Forward Primer (5′-3′)	Reverse Primer (5′-3′)	Amplicon Size (bp)	Primer Position
MB157-1	F1: ATGGAAGCAATTGAGGAATC	R1: CCGATGAACCTGCTCTTGTG	243	1st. Exon
MB157-2	F2: CACAAGAGCAGGTTCATCGG	R2: GGATTTTTGGTTCAAACGGT	287	1st. Exon and Intron
MB157-3	F3: ACCGTTTGAACCAAAAATCC	R3: TACAATGACCTAGTACTCCC	243	1st. Intron
MB157-4	F4: GGGAGTACTAGGTCATTGTA	R4: TCCAAGAAGTCGAGAGAGTA	790	1st. Intron and 2nd. Exon
MB157-5	F5: TACTCTCTCGACTTCTTGGA	R5: TATGTCGAAAGCATCTCTTC	782	2nd. Exon
MB157-6	F6: GAAGAGATGCTTTCGACATA	R6: TTCAATGATTGGCGACACTG	805	2nd. Exon to 4th. Exon

## Supplementary Materials

The following are available online at https://www.mdpi.com/2073-4425/11/2/220/s1; Table S1: Details of 22 melon accessions used and their disease response, as determined by bioassay and PCR-based assay using polymorphic InDel marker MB157-2. Table S2: Comparison of *Acidovorax citrulli* bioassay results and InDel marker BM157-2 bands for the putative R-gene MELO3C022157 in a population of 491 F2 melon individuals raised from resistant and susceptible parental accessions PI 353814 and PI 614596, respectively. ”+”, ”ᴎ” and ”-“ indicate resistant, heterozygous and susceptible bands for the InDel marker BM157-2. Figure S1: Disease scores used to assess the severity of bacterial fruit blotch (BFB) caused by *A. citrulli* at 12 days after inoculation. Scores range from 1 to 6, with 1 representing leaves with no damage and 6 representing maximum damage. All leaves were detached just before photographs were taken. Figure S2: Disease symptoms on resistant (R) and susceptible (S) melon parental accessions PI 353814 and PI 614596, respectively, their F1 hybrid and 491 individuals of the F2 population at 12 days after inoculation with *A. citrulli*. All leaves were detached just before photographs were taken. Figure S3: Frequency distribution of BFB scores in melon P_1_, P_2_, F_1_ and F_2_ populations. Figure S4: Detection of length polymorphism in 57 putative disease resistance genes containing NBS, LRR, CC and TIR domains by PCR-based assay. Details of the genes and corresponding primer specifications are presented in Table 1. Genes 40-2 and 57 were not amplified. Gene 41 (red underline) showed conspicuous length polymorphism. R—resistant parent PI 353814, S—susceptible parent PI 614596 and F1—hybrid (PI 614596 × PI 353814). Figure S5: (a) Bacterial fruit blotch (BFB) disease symptoms on 22 melon accessions at 12 days after inoculation with *A. citrulli*. Details of these 22 melon accessions are shown in Supplementary Table S1. All leaves were detached just before photographs were taken. (b) Validation of resistance status to BFB using InDel marker MB157-2 in the 22 melon accessions. R—resistant (PI 353814) and S—susceptible (PI 614596) used as control. Figure S6: Sequences and alignments of cloned and sequenced TIR-NBS-LRR gene MELO3C022157 from resistant (PI353814) and susceptible (PI614596) melon accessions. (a) Genomic sequences of reference, resistant and susceptible accessions. (b) Their alignment with reference sequences retrieved from the Cucurbit Genomics database (http://cucurbitgenomics.org) considering DHL92 as the reference genome. In the sequence alignment, asterisks (*) indicate sequence similarity and absence of asterisks indicates sequence dissimilarity; em-dash (—) and green color designate insertion/deletion of nucleotides. In the gDNA sequences, grey highlights illustrate the position of exons, yellow highlights indicate InDel primer (MB157-2-F and MB157-2-R) sequences and light blue indicates the primer name and InDel position. (c) Translated protein sequences of reference gene, resistant and susceptible parent sequences determined using the “Translate Tool-ExPASy” (https://web.expasy.org/translate/) web-based database and showing amino acid alterations and truncated protein in the susceptible parent.
