# Supplementary material for: Development of Molecular Marker Linked with Bacterial Fruit Blotch Resistance in Melon (Cucumis melo L.)
Source: Genes (Basel). 2020 Feb 19;11(2):220. doi: 10.3390/genes11020220 (PMC7074460; doi:10.3390/genes11020220)
Supplement: Supplementary file 1 [file genes-11-00220-s001.pdf]

Supplementary Information

Development of Molecular Marker linked with Bacterial Fruit Blotch resistance in Melon (*Cucumis melo* L.)

Md. Rafiqul Islam[1,2], Mohammad Rashed Hossain[1,3], Denison Michael Immanuel Jesse[1], Hee-Jeong Jung[1], Hoy-Taek Kim[1*], Jong-In Park[1] and Ill-Sup Nou[1*]

[1]Department of Horticulture, Sunchon National University, Suncheon, Jeonnam, 57922, Republic of Korea
[2]Depatmment of Biotechnology, Sher-e-Bangla Agricultural University, Dhaka-1207, Bangladesh
[3]Department of Genetics and Plant Breeding, Bangladesh Agricultural University, Mymensing-2202, Bangladesh

rafiqul@sau.edu.bd (MRI); m.r.hossain@bau.edu.bd (MRH); michaelijesse@gmail.com (DMIJ); gml79wjd@sunchon.ac.kr(H.-J.J.); htkim@sunchon.ac.kr (H.-T. K.); jipark@sunchon.ac.kr (J.-I.P.); nis@sunchon.ac.kr (I.-S. N.)

[*]**Correspondence:** htkim@sunchon.ac.kr (H-TK.); nis@sunchon.ac.kr (I-SN), Tel.: +82-61-750-3249 (I-SN); Fax: +82-61-750-5389 (I-SN)

Table S1: Details of 22 melon accessions used and their disease response, as determined by bioassay and PCR-based assay using polymorphic InDel marker MB157-2.

Sl.	Genotype	Taxonomy	Origin	Bioassay: Percent infected area per leaf	Bioassay: Phenotype	Genotype (InDel marker MB157-2)
**1**	PI 353814	*Cucumis melo* subsp. *melo*	Israel	0	Resistant (Control)	+
**2**	PI 614596	*Cucumis melo* subsp. *melo*	Madhya Pradesh, India	93	Susceptible (Control)	-
3	PI 140471	*Cucumis melo*	USA	3	Resistant	+
4	PI 420145	*Cucumis melo*	USA	5	Resistant	+
5	PI 614525	*Cucumis melo* subsp. *agrestis*	Madhya Pradesh, India	86	Susceptible	-
6	PI 482398	*Cucumis melo* subsp. *melo*	Zimbabwe	54	Susceptible	-
7	PI482399	*Cucumis melo* subsp. *melo*	USA	48	Susceptible	-
8	PI 536473	*Cucumis melo* subsp. *agrestis*	Maldives	39	Susceptible	-
9	PI 504558	*Cucumis ficifolius*	India	47	Susceptible	-
10	PI 343701	*Cucumis pustulatus* (Wild)	Nigeria	35	Susceptible	-
11	PI 614401	*Cucumis melo* subsp. *melo*	Rajasthan, India	24	Susceptible	-
12	PI 614601	*Cucumis melo* subsp. *melo*	Rajasthan, India	49	Susceptible	-
13	PI 147065	*Cucumis anguria var. longaculeatus* (Wild)	Amazonas, Brazil	28	Susceptible	-
14	PI 157076	*Cucumis melo* subsp. *melo*	China	44	Susceptible	-
15	PI 157082	*Cucumis melo* subsp. *melo*	Cornell University, USA	37	Susceptible	-
16	CornellZPPM	*Cucumis melo*	Cornel University, USA	48	Susceptible	-
17	PI 357152	*Cucumis melo* subsp. *melo*	Peru, Tacna	36	Susceptible	-
18	PI 378171	*Cucumis melo* subsp. *melo*	Israel	55	Susceptible	-
19	PI 381763	*Cucumis melo* subsp. *melo*	India, Rajasthan	50	Susceptible	-
20	PI 422164	*Cucumis melo* subsp. *melo*	Czech Republic	72	Susceptible	-

21	PI 504531	*Cucumis melo* subsp. *melo*	Korea, South	39	Susceptible	-
22	PI 512417	*Cucumis melo* subsp. *melo*	Spain, Cordoba	31	Susceptible	-
23	PI 512568	*Cucumis melo*	Spain, Castellon de Plana	64	Susceptible	-
24	SCNU1154	*Cucumis melo*	SCNU, South Korea	48	Susceptible	-

Resistant (PI 353814) and susceptible parent (PI 614596) were included as control in both bioassay and PCR based assay. All landraces/inbred were collected form USDA, USA, except SCNU1154 which was collected from South Korea. '+' and '-' indicate resistant and susceptible bands for the InDel marker BM157-2.

Table S2: Comparison of *Acidovorax citrulli* bioassay results and InDel marker BM157-2 bands for the putative R-gene MELO3C022157 in a population of 491 F2 melon individuals raised from resistant and susceptible parental accessions PI 353814 and PI 614596, respectively. ”+”, ”и” and ”-“ indicate resistant, heterozygous and susceptible bands for the InDel marker BM157-2.

Sl.	PIA/L	P	G	Sl.	PIA/L	P	G	Sl.	PIA/L	P	G	Sl.	PIA/L	P	G	Sl.	PIA/L	P	G
1	46	S	-	45	97	S	-	89	45	S	-	133	8	R	и	177	0	R	и
2	0	R	+	46	10	R	+	90	5	R	и	134	0	R	+	178	42	S	-
3	2	R	и	47	5	R	+	91	3	R	+	135	0	R	и	179	77	S	-
4	39	S	-	48	0	R	+	92	9	R	+	136	3	R	и	180	0	R	и
5	31	S	-	49	100	S	-	93	6	R	и	137	4	R	и	181	38	S	-
6	53	S	-	50	2	R	+	94	14	R	и	138	0	R	+	182	7	R	и
7	1	R	и	51	8	R	и	95	5	R	и	139	44	S	и	183	41	S	-
8	3	R	и	52	29	S	и	96	4	R	и	140	0	R	и	184	3	R	+
9	6	R	+	53	4	R	и	97	11	R	и	141	6	R	+	185	5	R	и
10	4	R	+	54	16	R	и	98	74	S	-	142	0	R	и	186	0	R	+
11	9	R	+	55	43	S	-	99	7	R	и	143	3	R	+	187	0	R	и
12	44	S	-	56	5	R	и	100	38	S	-	144	32	S	и	188	53	S	-
13	17	R	и	57	12	R	+	101	45	S	-	145	2	R	и	189	3	R	и
14	5	R	и	58	24	S	-	102	0	R	и	146	0	R	и	190	64	S	и
15	7	R	+	59	5	R	+	103	33	S	-	147	5	R	+	191	6	R	и
16	3	R	+	60	9	R	и	104	2	R	и	148	7	R	и	192	3	R	и
17	11	R	и	61	4	R	и	105	0	R	+	149	4	R	+	193	0	R	+
18	29	S	-	62	0	R	и	106	27	S	-	150	9	R	+	194	0	R	и
19	5	R	и	63	27	S	-	107	4	R	и	151	0	R	и	195	56	S	-
20	14	R	и	64	3	R	и	108	3	R	+	152	10	R	и	196	28	S	-
21	33	S	-	65	5	R	и	109	15	R	+	153	13	R	и	197	23	S	-
22	3	R	и	66	59	S	-	110	61	S	-	154	18	R	и	198	41	S	-
23	2	R	+	67	15	R	+	111	25	S	-	155	4	R	и	199	50	S	-
24	6	R	и	68	7	R	и	112	0	R	и	156	0	R	+	200	0	R	и
25	10	R	и	69	12	R	и	113	2	R	и	157	6	R	и	201	4	R	+
26	52	S	-	70	6	R	и	114	46	S	-	158	30	S	-	202	22	S	-
27	0	R	+	71	11	R	и	115	7	R	+	159	0	R	и	203	29	S	-
28	4	R	и	72	38	S	-	116	23	S	-	160	73	S	-	204	33	S	-
29	71	S	-	73	52	S	-	117	9	R	и	161	0	R	и	205	46	S	-
30	5	R	и	74	32	S	-	118	3	R	+	162	0	R	и	206	5	R	+
31	3	R	и	75	0	R	и	119	0	R	+	163	5	R	и	207	26	S	-
32	49	S	+	76	6	R	и	120	4	R	и	164	0	R	+	208	4	R	и
33	10	R	+	77	2	R	+	121	75	S	-	165	2	R	и	209	3	R	+
34	3	R	+	78	5	R	+	122	5	R	+	166	4	R	+	210	12	R	и
35	5	R	+	79	9	R	+	123	83	S	-	167	0	R	и	211	5	R	и
36	13	R	+	80	4	R	+	124	0	R	+	168	3	R	и	212	30	S	-
37	2	R	+	81	30	S	-	125	0	R	и	169	0	R	и	213	0	R	и
38	0	R	и	82	7	R	и	126	8	R	и	170	4	R	+	214	24	S	-
39	7	R	и	83	3	R	и	127	2	R	+	171	62	S	-	215	6	R	и
40	53	S	-	84	0	R	+	128	3	R	и	172	43	S	-	216	10	R	и
41	64	S	-	85	13	R	и	129	0	R	+	173	8	R	и	217	8	R	+
42	76	S	-	86	98	S	-	130	6	R	+	174	34	S	-	218	5	R	и
43	3	R	и	87	39	S	-	131	8	R	и	175	47	S	-	219	27	S	-
44	61	S	-	88	9	R	+	132	0	R	+	176	3	R	и	220	3	R	и

Sl.	PIA/L	P	G	Sl.	PIA/L	P	G	Sl.	PIA/L	P	G	Sl.	PIA/L	P	G	Sl.	PIA/L	P	G
221	6	R	и	277	0	R	+	333	5	R	и	389	5	R	и	445	2	R	и
222	0	R	+	278	9	R	+	334	51	R	+	390	0	R	и	446	29	S	-
223	5	R	+	279	36	S	-	335	25	S	-	391	0	R	+	447	50	S	-
224	9	R	+	280	6	R	и	336	3	R	и	392	0	R	и	448	27	S	-
225	5	R	и	281	4	R	+	337	65	S	-	393	0	R	и	449	53	S	-
226	6	R	+	282	12	R	и	338	77	S	-	394	6	R	+	450	0	R	и
227	3	R	и	283	84	S	-	339	27	S	-	395	37	S	+	451	4	R	и
228	4	R	+	284	0	R	+	340	6	R	+	396	4	R	+	452	0	R	+
229	0	R	+	285	5	R	и	341	3	R	и	397	0	R	и	453	0	R	и
230	13	R	+	286	0	R	+	342	34	S	-	398	0	R	и	454	24	S	-
231	4	R	и	287	31	S	-	343	0	R	и	399	4	R	и	455	66	S	-
232	0	R	+	288	24	S	-	344	23	S	-	400	0	R	+	456	4	R	и
233	33	S	и	289	5	R	и	345	35	S	-	401	0	R	и	457	38	S	-
234	0	R	и	290	8	R	+	346	2	R	и	402	0	R	и	458	31	S	-
235	7	R	+	291	3	R	и	347	25	S	-	403	0	R	и	459	0	R	и
236	9	R	+	292	0	R	+	348	0	R	и	404	0	R	+	460	35	S	-
237	10	R	и	293	6	R	и	349	23	S	-	405	5	R	+	461	24	S	-
238	0	R	и	294	78	S	-	350	0	R	и	406	7	R	и	462	0	R	и
239	11	R	+	295	4	R	и	351	5	R	+	407	0	R	и	463	0	R	и
240	14	S	-	296	0	R	+	352	7	R	и	408	11	R	и	464	36	S	-
241	8	R	и	297	24	S	-	353	0	R	+	409	5	R	+	465	56	S	-
242	5	R	+	298	73	S	-	354	9	R	и	410	0	R	и	466	2	R	+
243	9	R	и	299	0	R	и	355	39	S	+	411	0	R	и	467	24	S	-
244	5	R	+	300	6	R	и	356	0	R	+	412	5	R	и	468	47	S	-
245	2	R	+	301	35	S	-	357	0	R	и	413	3	R	и	469	0	R	и
246	14	R	и	302	5	R	и	358	4	R	+	414	0	R	и	470	5	R	и
247	0	R	+	303	57	S	-	359	6	H	+	415	47	S	-	471	0	R	и
248	8	R	и	304	10	R	и	360	2	R	и	416	0	R	и	472	0	R	и
249	12	R	и	305	46	S	-	361	0	R	+	417	3	R	и	473	0	R	+
250	5	R	и	306	74	S	-	362	10	R	и	418	0	R	и	474	0	R	и
251	0	R	+	307	0	R	и	363	5	R	и	419	4	R	+	475	0	R	и
252	34	S	+	308	0	R	+	364	13	R	и	420	0	R	и	476	3	R	и
253	3	R	и	309	0	R	и	365	4	R	+	421	0	R	и	477	0	R	и
254	6	R	+	310	62	S	-	366	37	S	+	422	5	R	и	478	5	R	+
255	0	R	+	311	6	R	+	367	0	R	и	423	40	S	-	479	8	R	+
256	4	R	и	312	0	R	и	368	6	R	и	424	0	R	-	480	69	S	-
257	3	R	и	313	31	S	-	369	0	R	+	425	35	S	-	481	0	R	и
258	4	R	и	314	0	R	и	370	7	R	+	426	30	S	-	482	72	S	-
259	24	R	и	315	48	S	-	371	5	R	и	427	7	R	и	483	0	R	+
260	0	R	+	316	8	R	+	372	6	R	+	428	6	R	+	484	3	R	+
261	24	S	-	317	26	S	-	373	7	R	и	429	0	R	и	485	0	R	+
262	51	S	-	318	39	S	-	374	5	R	+	430	8	R	и	486	0	R	+
263	62	S	-	319	22	S	-	375	0	R	и	431	4	R	и	487	0	R	+
264	43	S	-	320	15	R	+	376	9	R	+	432	31	S	-	488	33	S	-
265	6	R	+	321	36	S	-	377	0	R	+	433	35	S	-	489	5	R	+
266	22	S	-	322	82	S	-	378	0	R	и	434	3	R	и	490	8	R	и
267	25	S	-	323	12	R	и	379	4	R	+	435	6	R	и	491	54	S	-
268	30	S	-	324	10	R	и	380	0	R	и	436	0	R	и				
269	43	S	-	325	3	R	+	381	4	R	+	437	9	R	и				
270	7	R	+	326	5	R	+	382	0	R	+	438	0	R	+				
271	11	R	и	327	34	S	-	383	6	R	и	439	2	R	и				
272	28	S	-	328	0	R	и	384	3	R	и	440	0	R	и				
273	10	R	и	329	3	R	+	385	8	R	+	441	0	R	и				
274	0	R	+	330	7	R	и	386	0	R	и	442	0	R	+				
275	4	R	и	331	9	R	и	387	0	R	+	443	4	R	+				
276	0	R	и	332	32	S	-	388	5	R	и	444	5	R	и				

PIA/L. Percent infected Area per leaf, P. phenotype and G. Genotype. Red text indicate mismatch between bioassay phenotype and marker assisted genotype.

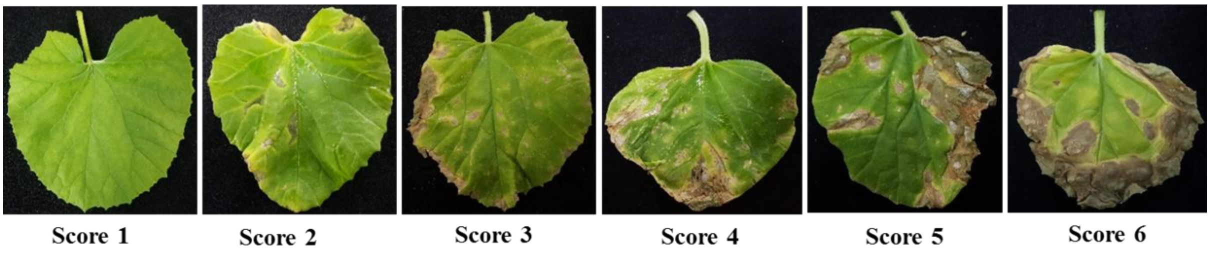

Figure S1: Disease scores used to assess the severity of bacterial fruit blotch (BFB) caused by *A. citrulli* at 12 days after inoculation. Scores range from 1 to 6, with 1 representing leaves with no damage and 6 representing maximum damage. All leaves were detached just before photographs were taken.

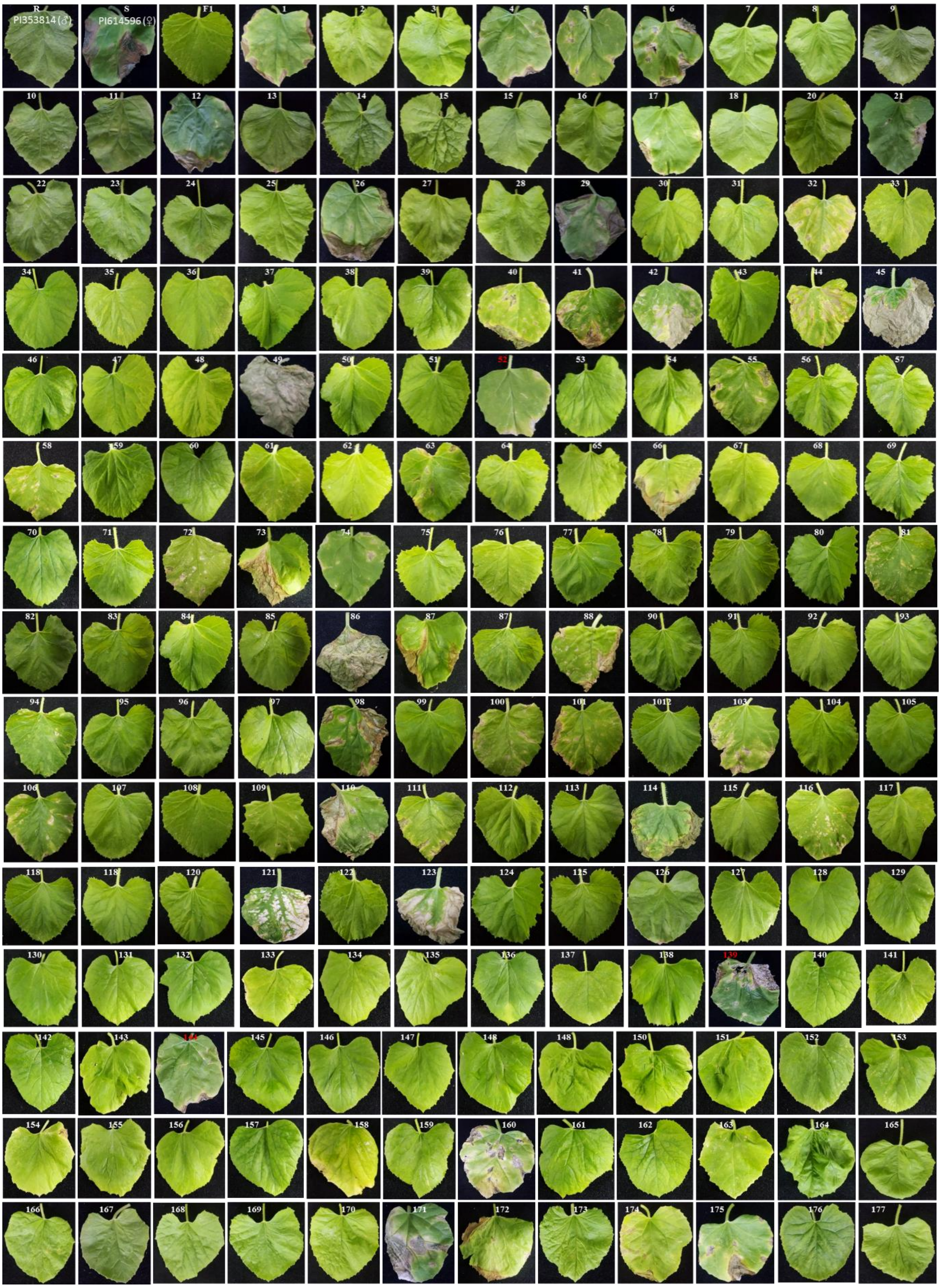

Fig. S1. Continued

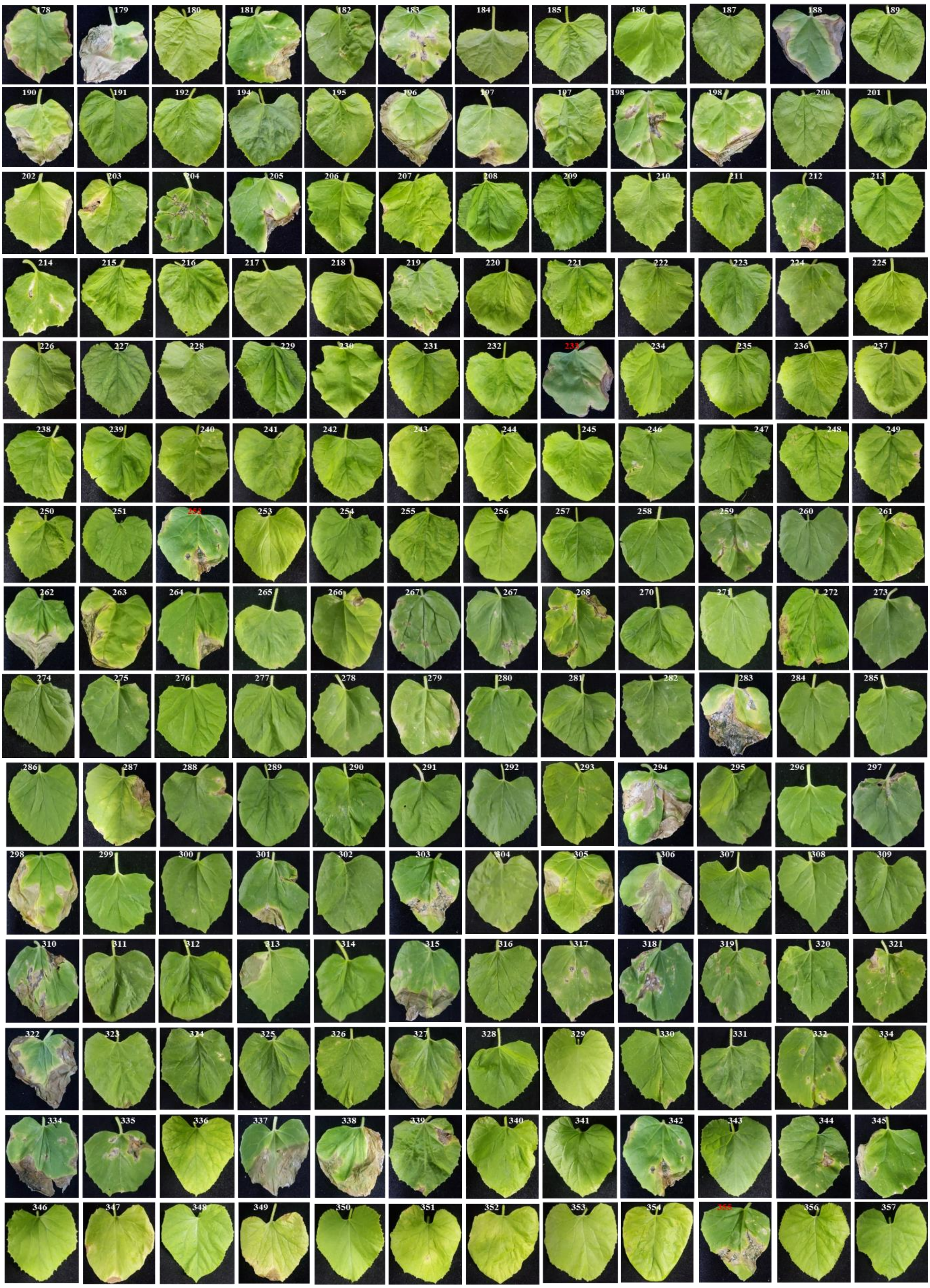

Fig S1. Continued

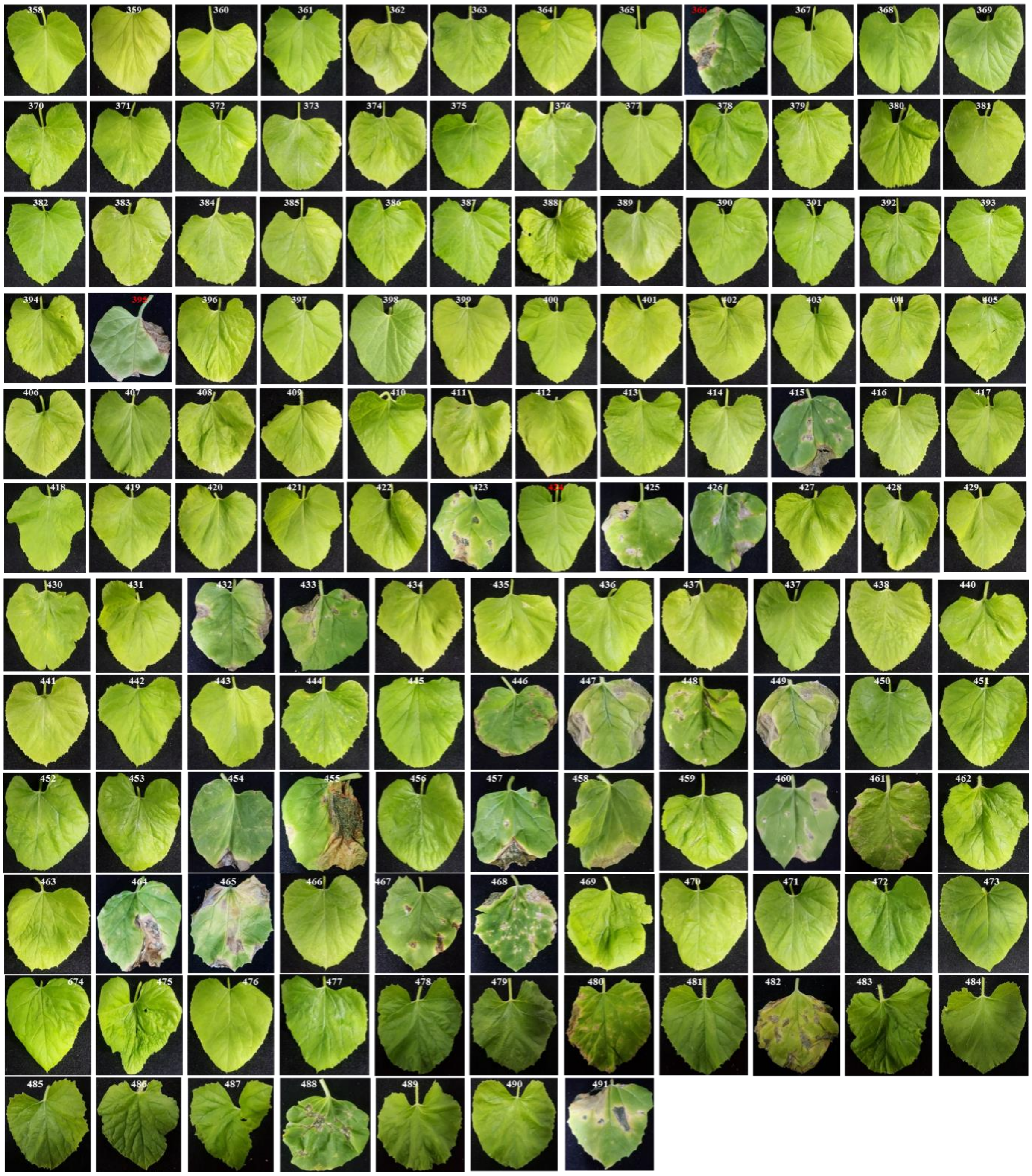

Figure S2: Disease symptoms on resistant (R) and susceptible (S) melon parental accessions PI 353814 and PI 614596, respectively, their F1 hybrid and 491 individuals of the F2 population at 12 days after inoculation with *A. citrulli*. All leaves were detached just before photographs were taken.

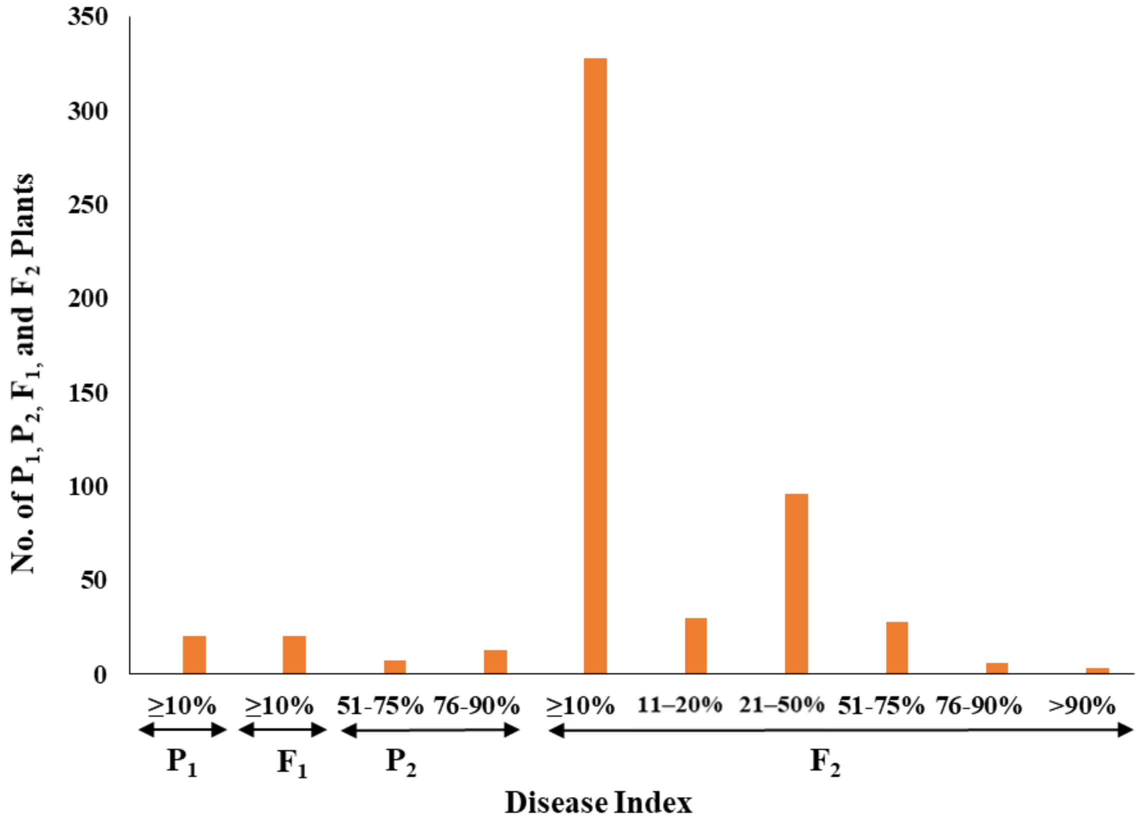

Figure S3: Frequency distribution of BFB scores in melon P_1, P_2, F_1 and F_2 populations.

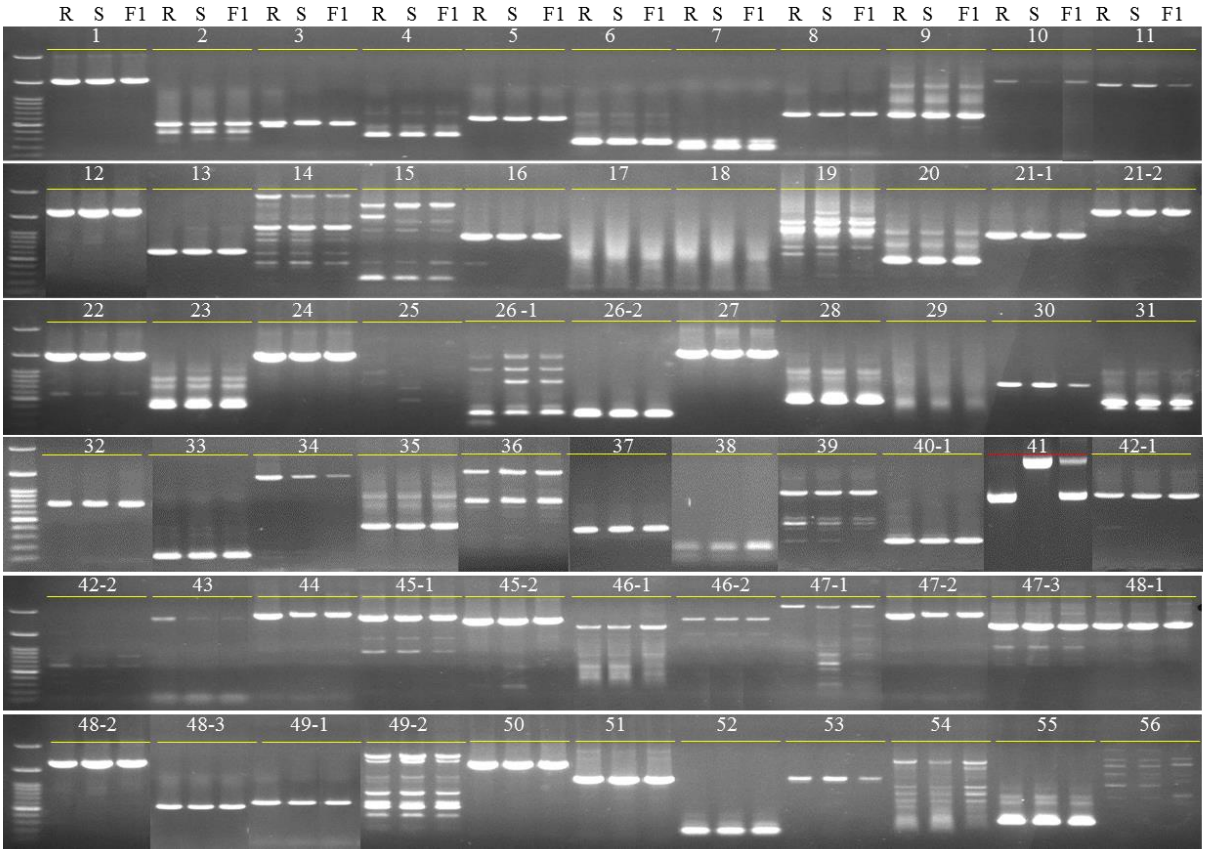

Figure S4: Detection of length polymorphism in 57 putative disease resistance genes containing NBS, LRR, CC and TIR domains by PCR-based assay. Details of the genes and corresponding primer specifications are presented in Table 1. Genes 40-2 and 57 were not amplified. Gene 41 (red underline) showed conspicuous length polymorphism. R—resistant parent PI 353814, S—susceptible parent PI 614596 and F1—hybrid (PI 614596 × PI 353814).

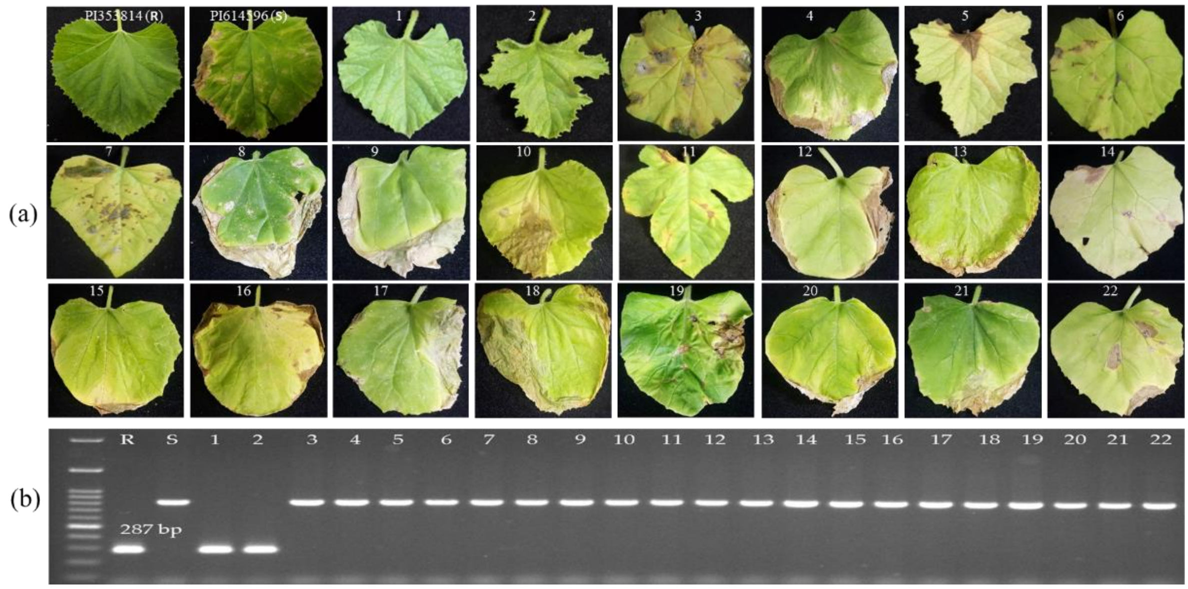

Figure S5: (a) Bacterial fruit blotch (BFB) disease symptoms on 22 melon accessions at 12 days after inoculation with *A. citrulli*. Details of these 22 melon accessions are shown in Supplementary Table S1. All leaves were detached just before photographs were taken. (b) Validation of resistance status to BFB using InDel marker MB157-2 in the 22 melon accessions. R—resistant (PI 353814) and S—susceptible (PI 614596) used as control.

a. Genomic DNA (gDNA) sequence (with intron)

>MELO3C022157Ref
ATGGAAGCAATTGAGGAATCAAGAACAGCTATTGTGGTTTTATCACAAAACTATTCTACTTCAAGATGGTGCT
TGAGAGAATTGGAGAAGATTATGGAATCCATGGACGACGGAACAAATCGAGTTCTTCCTGTGTTTTACCATGT
AGATCCTTCTCATGTTCGTCATCAATCTGGACCTTTTGAGAGAAGCTTTGTTGAATATGAAAATAATGGACAAG
ACTCACAAGAGCAGGTTCATCGGTGGAGGGATGCTTTCGCTAGAGTTGGCCATCTTGCAGGGGTCGTAGTAAA
CAAAAACAGGTAGGGTCTCACTCTTCTCATTATCATTTTCTTCTTTTTTTTTTATATATAATTATTTCTTCGTATG
GTTAGATGTTAGGGAACAACAGACCTCCAAATATCTTCACAATAATATGATATTATCCACTTTAAACATACAT
CCTTCATGGTTTTCCTTTTGTTTTCACTCAAAAAACTATCTCATATATACCGTTTGAACCAAAAATCCATGGTCA
TCCCCTTAGTCTTATTTAACCGAAATGTGAAACTTTGATCGCTTTCTCACTTCGCATTAGACAAAATTGGGTGT
ACTCTAATACCATTTTTGAACCTAATAGTCTTTTGAGTTTTGTGCGTTCTGATACTATACGTTAGTGATAAACAT
CCTATTTTATCTCAAAAAACCAATTGACTAATGATCAGGAAGCTAGAGGGAGTACTAGGTCATTGTATCTTAT
CAAACATGCATTTATGAGGTCTCGAACTCCTTTGTTTTTTCCAATGTAGCACACAACCTAGCTCAACACACTTT
TATTTTTTAGCATTCAAGTACATAAAATAATATGCGAATAGAAGAAGTTAGAGATTCCTAAATTAATAGCCTA
ATAAATAGTACTTTAAAAAAATAATATTACACATAATTGTTTCTTTACATATTCCTAGAATCTCTGCTTGTTCTA
AAAAGTCTCATGGAATATATAGAAAGGAGCCCGACTAATTGAAGAATTAGATACACAAACTTTTTTAGAATTA
GGAAAATAACTCTCTTCATTGATGGTGTGGATTATATAGTGGGGATTCTGTTAATAGTCGTTCATTGTATACTA
TAGGAAGAAAGAAAGCGAATCCAAGCACACCATAAATTTTTTTTTGACATACTACTCTTTGGCCATGCAGCCC
TGAAGTGGACAGTATCAACCGAATCACCAATCAAATATTTGATAAGTTGCGAAGACCTATGTTAATAGGCCCT
AATCAATTGAATTACTTGGTTGATATGCGAAGTAAGCTAAGGGATATCAATAACCTACTTGACTTTGAATCAG
ATGAAGTACGATTTATAGGAATAGTTGGAATGGGTGGTATTGGTAAAACAACTATTGCAAAAGTTTTACACGA
CAGTATTGCATTTACATTATTTGGTGAAAATTCTTGCTTTGTCACTATGTCTGGGCGTGATATCGTCACGGTCC
AATGTCTACTACTCTCTCGACTTCTTGGAACTAGGGAGAATATTAACATTTTAGAAAAGAATGAAGGAGCAAA
CATGATTAAAGATTGTTTGAGTAGGAGAAAGGTTTTGATTATTTTTGATGGGGTGAATGATAGAGAGGAATTA
GGATACATAGCCGGAAGTTTTGATTGGTTTGGTCGAGGAAGTCGAGTCATCATTACCACTAGAAATAAAAATG
TTTTTTCTCACCCCAATCATGAACAAGTTCAACTCTACAATGTGAAACCACTTGATTACAACACTTCATTCTCA
CTTTTTTGGAAGCATGCATTTGATCAACAAAGTGGGGGTCCAAGTGAACAACAATTCATACAACTTAGTCAGA
ATATAGTGGAAAAGGTCGAAGGAAATCCACAAGCATTGGAACAAATTGGATCATTTTTGCGTGGTAAAGATA
TTAATGTATGGAAAGAAGAATTGAAGAGCCTTGTTTTAGTTGATAATGAACGTCTCTTCAAAATATTAAAGAT
AAGTTTTGATCAATTAGGGACAAAAGGCCAACAAGCTTTTCTTGATTTGGCATGTTTCTTCAATGGAAAAAGT
ACAGGCAAAATTATTGAAATACTTGCGAGTTTAGAATACAATTCCCCCAGCGAAGTACTAAAGTTGTTGTGTG
ATAGATATCTTATTGAAATTAGAGATGGAGACACAGTATGTATGCCCAATTTGATACAAGAAATGGGTCGAGA
AATAGAACGAAAAAAACGTCAAAGAAGCAGGATTTGGCTTAGAAGAGATGCTTTCGACATATTTGATGAACA
ACATGTAAGTCTATATATGTTACATTATATTATTGTACATTATTTTCCATGTTATTTAATTATATTAACTGATTT
ACTTTGGCAGGGAGTAAAAGACATAAAAGGTGTTGTGTTGGACAAGAGAGACACAGAACCAAACTTAAAGTT
GAAGGCTAAACAATTACAAGATATGAGCCGTTTAAAAATATTAGAGATTGACAATGTGCAGCTGAGTCCAAG
AAATCAAAATGATCTCTCAAATCAGCTTCGATTGCTCCACTGGGATGGCTTTCCTTCAGACACTTTGCCACTAA
ATTTCGAAGCACCATATTTATTTGAACTTCTCTTGCCTAATGCACAAACCACTCATCTTTGGAAAGAACTAAAG
GTTAGACATATATTTGCAAATACTTTAACCTGCGCTATATATATATATATGTTGTGATTGTGCTATTTGCAATA
ATTTTTCAAATTTTCTTTCATAACATATATAAATACATGTTTTGTTATTTGGTAACAGGGATTTAAGAAATTAA
AGGTAATCGATGTTAGCAATTCCCAAACTTTGGTGGAGACACCGAATTTGAGTGCTGTTCCAAATCTAGAAAG
ATTGATTCTATGTAATTGTACAAGATTGAAGAAAATTGATAATTCAATTACAAAATTGAGACTTCTAGTTTTAG
TAGACCTCACAGGCTGTGTTCGCCTCGAAACATCCGAGTGCATCGATATCTTGAAGAGTCGCCCAACAGTAGA
ACTTCGTGGCTTAGTTCTACAGTGTCGCCAATCATTGAAAGGTATTTGTTATATTCGTAAATTTTTTCACTTCTT
TTTTAAAATTCAGGATCATTTTGTGTAA

>PI353814_R
ATGGAAGCAATTGAGGAATCAAGAACAGCTATTGTGGTTTTATCACAAAACTATTCTACTTCAAGATGGTGCT
TGAGAGAATTGGAGAAGATTATGGAATCCATGGACGACGGAACAAATCGAGTTCTTCCTGTGTTTTACCATGT
AGATCCTTCTCATGTTCGTCATCAATCTGGACCTTTTGAGAGAAGCTTTGTTGAATATGAAAATAATGGACAAG
ACTCACAAGAGCAGGTTCATCGGTGGAGGGATGCTTTCGCTAGAGTTGGCCATCTTGCAGGGGTCGTAGTAAA
CAAAAACAGGTAGGGTCTCACTCTTCTCATTATCATTTTCTTCTTTTTTTTTTATATATAATTATTTCTTCGTATG
GTTAGATGTTAGGGAACAACAGACCTCCAAATATCTTCACAATAATATGATATTATCCACTTTAAACATACAT
CCTTCATGGTTTTCCTTTTGTTTTCACTCAAAAAACTATCTCATATATACCGTTTGAACCAAAAATCCATGGTCA
TCCCCTTAGTCTTATTTAACCGAAATGTGAAACTTTGATCGCTTTCTCACTTCGCATTAGACAAAATTGGGTGT
ACTCTAATACCATTTTTGAACCTAATAGTCTTTTGAGTTTTGTGCGTTCTGATACTATACGTTAGTGATAAACAT
CCTATTTTATCTCAAAAAACCAATTGACTAATGATCAGGAAGCTAGAGGGAGTGTCATTGTATCTTATCAAAC
ATGCATTTATGAGGTCTCGAACTCCTTTGTTTTTTCCAATGTAGCACACAACCTAGCTCAACACACTTTTATTTT
TTAGCATTCAAGTACATAAAATAATATGCGAATAGAAGAAGTTAGAGATTCCTAAATTAATAGCCTAATAAAT
AGTACTTTAAAAAAATAATATTACACATAATTGTTTCTTTACATATTCCTAGAATCTCTGCTTGTTCTAAAAAG
TCTCATGGAATATATAGAAAGGAGCCCGACTAATTGAAGAATTAGATACACAAACTTTTTTAGAATTAGGAAA
ATAACTCTCTTCATTGATGGTGTGGATTATATAGTGGGGATTCTGTTAATAGTCGTTCATTGTATACTATAGGA
AGAAAGAAAGCGAATCCAAGCACACCATAAATTTTAGTTTGACATACTACTCTTTGGCCATGCAGCCCTGAAG

TGGACAGTATCAACCGAATCACCAATCAAATATTTGATAAGTTGCGAAGACCTATGTTAATAGGCCCTAATCA
ATTGAATTACTTGGTTGATATGCGAAGTAAGCTAAGGGATATCAATAACCTACTTGACTTTGAATCAGATGAA
GTACGATTTATAGGAATAGTTGGAATGAGTGGTATTGGTAAAACAACTATTGCAAAAGTTTTACACGACAGTA
TTGCATTTACATTATTTGGTGAAAATTCTTGCTTTGTCACTATGTCTGGGCGTGATATCGTCACGGTCCAATGTC
TACTACTCTCTCGACTTCTTGGAACTAGGGAGAATATTAACATTTTAGAAAAGAATGAAGGAGCAAACATGAT
TAAAGATTGTTTGAGTAGGAGAAAGGTTTTGATTATTTTTGATGGGGTGAATGATAGAGAGGAATTAGGATAC
ATAGCCGGAAGTTTTGATTGGTTTGGTCGAGGAAGTCGAGTCATCATTACCACTAGAAATAAAAATGTTTTTTC
TCACCCCAATCATGAACAAGTTCAACTCTACAATGTGAAACCACTTGATTACAACACTTCATTCTCACTTTTTT
GGAAGCATGCATTTGATCAACAAAGTGGGGGTCCAAGTGAACAACAATTCATACAACTTAGTCAGAATATAG
TGGAAAAGGTCGAAGGAAATCCACAAGCATTGGAACAAATTGGATCATTTTTGCGTGGTAAAGATATTAATGT
ATGGAAAGAAGAATTGAAGAGCCTTGTTTTAGTTGATAATGAACGTCTCTTCAAAATATTAAAGATAAGTTTT
GATCAATTAGGGACAAAAGGCCAACAAGCTTTTCTTGATTTGGCATGTTTCTTCAATGGAAAAAGTACAGGCA
AAATTATTGAAATACTTGCGAGTTTAGAATACAATTCCCCCAGCGAAGTACTAAAGTTGTTGTGTGATAGATA
TCTTATTGAAATTAGAGATGGAGACACAGTATGTATGCCCAATTTGATACAAGAAATGGGTCGAGAAATAGA
ACGAAAAAAACGTCAAAGAAGCAGGATTTGGCTTAGAAGAGATGCTTTCGACATATTTGATGAACAACATGT
AAGTCTATATATGTTACATTATATTATTGTACATTATTTTCCATGTTATTTAATTATATTAACTGATTTACTTTG
GCAGGGAGTAAAAGACATAAAAGGTGTTGTGTTGGACAAGAGAGACACAGAACCAAACTTAAAGTTGAAGG
CTAAACAATTACAAGATATGAGCCGTTTAAAAATATTAGAGATTGACAATGTGCAGCTGAGTCCAAGAAATCA
AAATGATCTCTCAAATCAGCTTCGATTGCTCCACTGGGATGGCTTTCCTTCAGACACTTTGCCACTAAATTTCG
AAGCACCATATTTATTTGAACTTCTCTTGCCTAATGCACAAACCACTCATCTTTGGAAAGAACTAAAGGTTAGA
CATATATTTGCAAATACTTTAACCTGCGCTATATATATATATATATGTTGTGATTGTGCTATTTGCAATAATTTTTC
AAATTTTCTTTCATAACATATATAAATACATGTTTTGTTATTTGGTAACAGGGATTTAAGAAATTAAAGGTAAT
CGATGTTAGCAATTCCCAAACTTTGGTGGAGACACCGAATTTGAGTGCTGTTCCAAATCTAGAAAGATTGATT
CTATGTAATTGTACAAGATTGAAGAAAATTGATAATTCAATTACAAAATTGAGACTTCTAGTTTTAGTAGACCT
CACAGGCTGTGTTCGCCTCGAAACATCCGAGTGCATCGATATCTTGAAGAGTCGCCCAACAGTAGAACTTCGT
GGCTTAGTTCTACAGTGTCGCCAATCATTGAAAGGTATTTGTTATATTCGTAAATTTTTTCACTTCTTTTTTAAA
ATTCAGGATCATTTTGTGTAA

>PI614596_S
ATGGAAGCAATTGAGGAATCAAGAACAGCTATTGTGGTTTTATCACAAAACTATTCTACTTCAAGATGGTGCT
TGAGAGAATTGGAGAAGATTATGGAATCCATGGACGACGGAACAAATCGAGTTCTTCCTGTGTTTTACCATGT
AGATCCTTCTCATGTTCGTCATCAATCTGGACCTTTTGAGAGAAGCTTTGTTGAATATGAAAATAATGGACAAG
ACTCACAAGAGCAGGTTCATCGGTGGAGGGATGCTTTCGCTAGAGTTGGCCATCTTGCAGGGGTCGTAGTAAA
CAAAAACAGGTAGGGTCTCACTCTTCTCATTATCATTTTCTTCTTTTTTTTTTATATATAATTATTTCTTCGTATG
GTTAGATGTTAGGGAACAACAGACCTCCAAATATCTTCACAATAATATGATATTATCCACTTTAAACATACAT
CCTTCATGGTTTTCCGTTTCACTCCAAATTTAAGTTGGCATCTTGCAGGTCTTCTCATTATCATTTCTTCTTATAT
ATAATAAAACAACCAAAAACAACCAAAACCTTTCGCAGAGTTGAACCTTTCGCAGAGTTGGCATCTTGCAGGG
GTCGTAGTAAACAAACAACCAAAAACCTTTCGCAGAGTTGGCATCTTGCAGGAAACGGTAGGGTCTCACTCGC
AGAGTTGGCATCTTGCAGGTCTTCTCATTATCATTTCTTCTTTAGGGTCTCACTCGCAGAGTTGGCATCTTGCAG
GTCTTCTCATTATTTATATATAATTATTTCTTCGTATGGTTAGATTGTTTCACTCCAAAACAACCAAACCTTTCG
CAGAGTTGGCATCTTGCAGGGGTCGTGTTAGGGAACAACAGAGGTCGTAGTAAACAAAAACGGTAGGGTCTC
ACCTCCAAATATCTTCACAAGTAGGGTCTCATAATATGATATTATCCACTTTAAACATACATCCTTCATGGTTC
CTTTTGTTTTCACTCAAAAAACTATCTCATATATACCGTTTGAACCAAAAATCCATGGTCATCCCCTTAGTCTTA
TTTAACCGAAATGTGAAACTTTGATCGCTTTCTCACTTCGCATTAGACAAAATTGGGTGTACTCTAATACCATT
TTTGAACCTAATAGTCTTTTGAGTTTTGTGCGTTCTGATACTATACGTTAGTGATAAACATCCTATTTTATCTCA
AAAAACCAATTGACTAATGATCAGGAAGCTAGAGGGAGTACTAGGTCATTGTATCTTATCAAACATGCATTTA
TGAGGTCTCGAACTCCTTTGTTTTTTCCAATGTAGCACACAACCTAGCTCAACACACTTTTATTTTTTAGCATTC
AAGTACATAAAATAATATGCGAATAGAAGAAGTTAGAGATTCCTAAATTAATAGCCTAATAAATAGTACTTTA
AAAAAATAATATTACACATAATTGTTTCTTTACATATTCCTAGAATCTCTGCTTGTTCTAAAAAGTCTCATGGA
ATATATAGAAAGGAGCCCGACTAATTGAAGAATTAGATACACAAACTTTTTTAGAATTAGGAAAATAACTCTC
TTCATTGATGGTGTGGATTATATAGTGGGGATTCTGTTAATAGTCGTTCATTGTATACTATAGGAAGAAAGAA
AGCGAATCCAAGCACACCATAAATTTTTTTTTGACATACTACTCTTTGGCCATGCAGCCCTGAAGTGGACAGTA
TCAACCGAATCACCAATCAAATATTTGATAAGTTGCGAAGACCTATGTTAATAGGCCCTAATCAATTGAATTA
CTTGGTTGATATGCGAAGTAAGCTAAGGGATATCAATAACCTACTTGACTTTGAATCAGATGAAGTACGATTT
ATAGGAATAGTTGGAATGGGTGGTATTGGTAAAACAACTATTGCAAAAGTTTTACACGACAGTATTGCATTTA
CATTATTTGGTGAAAATTCTTGCTTTGTCACTATGTCTGGGCGTGATATCGTCACGGTCCAATGTCTACTACTCT
CTCGACTTCTTGGAACTAGGGAGAATATTAACATTTTAGAATAGAATGAAGGAGCAAACATGATTAAAGATTG
TTTGAGTAGGAGAAAGGTTTTGATTATTTTTGATGGGGTGAATGATAGAGAGGAATTAGGATACATAGCCGGA
AGTTTTGATTGGTTTGGTCGAGGAAGTCGAGTCATCATTACCACTAGAAATAAAAATGTTTTTTCTCACCCCAA
TCATGAACAAGTTCAACTCTACAATGTGAAACCACTTGATTACAACACTTCATTCTCACTTTTTTGGAAGCATG

CATTTGATCAACAAAGTGGGGGTCCAAGTGAACAACAATTCATACAACTTAGTCAGAATATAGTGGAAAAGG
TCGAAGGAAATCCACAAGCATTGGAACAAATTGGATCATTTTTGCGTGGTAAAGATATTAATGTATGGAAAGA
AGAATTGAAGAGCCTTGTTTTAGTTGATAATGAACGTCTCTTCAAAATATGATAAGTTTTGATCAATTAGGGAC
AAAAGGCCAACAAGCTTTTCTTGATTTGGCATGTTTCTTCAATGGAAAAAGTACAGGCAAAATTATTGAAATA
CTTGCGAGTTTAGAATACAATTCCCCCAGCGAAGTACTAAAGTTGTTGTGTGATAGATATCTTATTGAAATTAG
AGATGGAGACACAGTATGTATGCCCAATTTGATACAAGAAATGGGTCGAGAAATAGAACGAAAAAAACGTCA
AAGAAGCAGGATTTGGCTTAGAAGAGATGCTTTCGACATATTTGATGAACAACATGTAAGTCTATATATGTTA
CATTATATTATTGTACATTATTTTCCATGTTATTTAATTATATTAACTGATTTACTTTGGCAGGGAGTAAAAGAC
ATAAAAGGTGTTGTGTTGGACAAGAGAGACACAGAACCAAACTTAAAGTTGAAGGCTAAACAATTACAAGAT
ATGAGCCGTTTAAGAATATTAGAGATTGACAATGTGCAGCTGAGTCCAAGAAATCAAAATGATCTCTCAAATC
AGCTTCGATTGCTCCACTGGGATGGCTTTCCTTCAGACACTTTGCCACTAAATTTCGAAGCACCATATTTATTT
GAACTTCTCTTGCCTAATGCACAAACCACTCATCTTTGGAAAGAACTAAAGGTTAGACATATATTTGCAAATA
CTTTAACCTGCGCTATATATATATATATATGTTGTGATTGTGCTATTTGCAATAATTTTTCAAATTTTCTTTCATAA
CATATATAAATACATGTTTTGTTATTTGGTAACAGGGATTTAAGAAATTAAAGGTAATCGATGTTAGCAATTCC
CAAACTTTGGTGGAGACACCGAATTTGAGTGCTGTTCCAAATCTAGAAAGATTGATTCTATGTAATTGTACAA
GATTGAAGAATATTGATAATTCAATTACAAAATTGAGACTTCTAGTTTTAGTAGACCTCACAGGCTGTGTTCGC
CTCGAAACATCCGAGTGCATCGATATCTTGAAGAGTCGCCCAACAGTAGAACTTCGTGGCTTAGTTCTACAGT
GTCGCCAATCATTGAAAGGTATTTGTTATATTCGTAAATTTTTTCACTTCTTTTTTAAAATTCAGGATCATTTTG
TGTAA

b. CLUSTALW multiple sequence alignment of gDNA sequence (with intron)

CLUSTAL 2.1 multiple sequence alignment

```
MELO3C022157Ref      ATGGAAGCAATTGAGGAATCAAGAACAGCTATTGTGGTTTTATCACAAAACTATTCTACT 60
PI353814_R           ATGGAAGCAATTGAGGAATCAAGAACAGCTATTGTGGTTTTATCACAAAACTATTCTACT 60
PI614596_S           ATGGAAGCAATTGAGGAATCAAGAACAGCTATTGTGGTTTTATCACAAAACTATTCTACT 60
                     ************************************************************

MELO3C022157Ref      TCAAGATGGTGCTTGAGAGAATTGGAGAAGATTATGGAATCCATGGACGACGGAACAAAT 120
PI353814_R           TCAAGATGGTGCTTGAGAGAATTGGAGAAGATTATGGAATCCATGGACGACGGAACAAAT 120
PI614596_S           TCAAGATGGTGCTTGAGAGAATTGGAGAAGATTATGGAATCCATGGACGACGGAACAAAT 120
                     ************************************************************

MELO3C022157Ref      CGAGTTCTTCCTGTGTTTTACCATGTAGATCCTTCTCATGTTCGTCATCAATCTGGACCT 180
PI353814_R           CGAGTTCTTCCTGTGTTTTACCATGTAGATCCTTCTCATGTTCGTCATCAATCTGGACCT 180
PI614596_S           CGAGTTCTTCCTGTGTTTTACCATGTAGATCCTTCTCATGTTCGTCATCAATCTGGACCT 180
                     ************************************************************

MELO3C022157Ref      TTTGAGAGAAGCTTTGTTGAATATGAAAATAATGGACAAGACTCACAAGAGCAGGTTCAT 240
PI353814_R           TTTGAGAGAAGCTTTGTTGAATATGAAAATAATGGACAAGACTCACAAGAGCAGGTTCAT 240
PI614596_S           TTTGAGAGAAGCTTTGTTGAATATGAAAATAATGGACAAGACTCACAAGAGCAGGTTCAT 240
                     ************************************************************

MELO3C022157Ref      CGGTGGAGGGATGCTTTCGCTAGAGTTGGCCATCTTGCAGGGGTCGTAGTAAACAAAAAC 300
PI353814_R           CGGTGGAGGGATGCTTTCGCTAGAGTTGGCCATCTTGCAGGGGTCGTAGTAAACAAAAAC 300
PI614596_S           CGGTGGAGGGATGCTTTCGCTAGAGTTGGCCATCTTGCAGGGGTCGTAGTAAACAAAAAC 300
                     ************************************************************

MELO3C022157Ref      AGGTAGGGTCTCACTCTTCTCATTATCATTTTCTTCTTTTTTTTTTATATATAATTATTT 360
PI353814_R           AGGTAGGGTCTCACTCTTCTCATTATCATTTTCTTCTTTTTTTTTTATATATAATTATTT 360
PI614596_S           AGGTAGGGTCTCACTCTTCTCATTATCATTTTCTTCTTTTTTTTTTATATATAATTATTT 360
                     ************************************************************

MELO3C022157Ref      CTTCGTATGGTTAGATGTTAGGGAACAACAGACCTCCAAATATCTTCACAATAATATGAT 420
PI353814_R           CTTCGTATGGTTAGATGTTAGGGAACAACAGACCTCCAAATATCTTCACAATAATATGAT 420
PI614596_S           CTTCGTATGGTTAGATGTTAGGGAACAACAGACCTCCAAATATCTTCACAATAATATGAT 420
                     ************************************************************

MELO3C022157Ref      ATTATCCACTTTAAACATACATCCTTCATGGTTTTCC----------------------- 457
PI353814_R           ATTATCCACTTTAAACATACATCCTTCATGGTTTTCC----------------------- 457
PI614596_S           ATTATCCACTTTAAACATACATCCTTCATGGTTTTCCGTTTCACTCCAAATTTAAGTTGG 480
                     *************************************

MELO3C022157Ref      ------------------------------------------------------------ 457
PI353814_R           ------------------------------------------------------------ 457
PI614596_S           CATCTTGCAGGTCTTCTCATTATCATTTCTTCTTATATATAATAAAACAACCAAAAACAA 540

MELO3C022157Ref      ------------------------------------------------------------ 457
PI353814_R           ------------------------------------------------------------ 457
PI614596_S           CCAAAACCTTTCGCAGAGTTGAACCTTTCGCAGAGTTGGCATCTTGCAGGGGTCGTAGTA 600

MELO3C022157Ref      ------------------------------------------------------------ 457
PI353814_R           ------------------------------------------------------------ 457
PI614596_S           AACAAACAACCAAAAACCTTTCGCAGAGTTGGCATCTTGCAGGAAACGGTAGGGTCTCAC 660

MELO3C022157Ref      ------------------------------------------------------------ 457
PI353814_R           ------------------------------------------------------------ 457
```

```
PI614596_S             TCGCAGAGTTGGCATCTTGCAGGTCTTCTCATTATCATTTCTTCTTTAGGGTCTCACTCG 720

MELO3C022157Ref        ------------------------------------------------------------ 457
PI353814_R             ------------------------------------------------------------ 457
PI614596_S             CAGAGTTGGCATCTTGCAGGTCTTCTCATTATTTATATATAATTATTTCTTCGTATGGTT 780

MELO3C022157Ref        ------------------------------------------------------------ 457
PI353814_R             ------------------------------------------------------------ 457
PI614596_S             AGATTGTTTCACTCCAAAACAACCAAACCTTTCGCAGAGTTGGCATCTTGCAGGGGTCGT 840

MELO3C022157Ref        ------------------------------------------------------------ 457
PI353814_R             ------------------------------------------------------------ 457
PI614596_S             GTTAGGGAACAACAGAGGTCGTAGTAAACAAAAACGGTAGGGTCTCACCTCCAAATATCT 900

MELO3C022157Ref        ------------------------------------------------------------ 457
PI353814_R             ------------------------------------------------------------ 457
PI614596_S             TCACAAGTAGGGTCTCATAATATGATATTATCCACTTTAAACATACATCCTTCATGGTTC 960

MELO3C022157Ref        -TTTTGTTTTCACTCAAAAAACTATCTCATATATACCGTTTGAACCAAAAATCCATGGTC 516
PI353814_R             -TTTTGTTTTCACTCAAAAAACTATCTCATATATACCGTTTGAACCAAAAATCCATGGTC 516
PI614596_S             CTTTTGTTTTCACTCAAAAAACTATCTCATATATACCGTTTGAACCAAAAATCCATGGTC 1020
                        ***********************************************************

MELO3C022157Ref        ATCCCCTTAGTCTTATTTAACCGAAATGTGAAACTTTGATCGCTTTCTCACTTCGCATTA 576
PI353814_R             ATCCCCTTAGTCTTATTTAACCGAAATGTGAAACTTTGATCGCTTTCTCACTTCGCATTA 576
PI614596_S             ATCCCCTTAGTCTTATTTAACCGAAATGTGAAACTTTGATCGCTTTCTCACTTCGCATTA 1080
                       ************************************************************

MELO3C022157Ref        GACAAAATTGGGTGTACTCTAATACCATTTTTGAACCTAATAGTCTTTTGAGTTTTGTGC 636
PI353814_R             GACAAAATTGGGTGTACTCTAATACCATTTTTGAACCTAATAGTCTTTTGAGTTTTGTGC 636
PI614596_S             GACAAAATTGGGTGTACTCTAATACCATTTTTGAACCTAATAGTCTTTTGAGTTTTGTGC 1140
                       ************************************************************

MELO3C022157Ref        GTTCTGATACTATACGTTAGTGATAAACATCCTATTTTATCTCAAAAAACCAATTGACTA 696
PI353814_R             GTTCTGATACTATACGTTAGTGATAAACATCCTATTTTATCTCAAAAAACCAATTGACTA 696
PI614596_S             GTTCTGATACTATACGTTAGTGATAAACATCCTATTTTATCTCAAAAAACCAATTGACTA 1200
                       ************************************************************

MELO3C022157Ref        ATGATCAGGAAGCTAGAGGGAGTACTAGGTCATTGTATCTTATCAAACATGCATTTATGA 756
PI353814_R             ATGATCAGGAAGCTAGAGGGAGT-----GTCATTGTATCTTATCAAACATGCATTTATGA 751
PI614596_S             ATGATCAGGAAGCTAGAGGGAGTACTAGGTCATTGTATCTTATCAAACATGCATTTATGA 1260
                       ***********************     ********************************

MELO3C022157Ref        GGTCTCGAACTCCTTTGTTTTTTCCAATGTAGCACACAACCTAGCTCAACACACTTTTAT 816
PI353814_R             GGTCTCGAACTCCTTTGTTTTTTCCAATGTAGCACACAACCTAGCTCAACACACTTTTAT 811
PI614596_S             GGTCTCGAACTCCTTTGTTTTTTCCAATGTAGCACACAACCTAGCTCAACACACTTTTAT 1320
                       ************************************************************

MELO3C022157Ref        TTTTTAGCATTCAAGTACATAAAATAATATGCGAATAGAAGAAGTTAGAGATTCCTAAAT 876
PI353814_R             TTTTTAGCATTCAAGTACATAAAATAATATGCGAATAGAAGAAGTTAGAGATTCCTAAAT 871
PI614596_S             TTTTTAGCATTCAAGTACATAAAATAATATGCGAATAGAAGAAGTTAGAGATTCCTAAAT 1380
                       ************************************************************

MELO3C022157Ref        TAATAGCCTAATAAATAGTACTTTAAAAAAATAATATTACACATAATTGTTTCTTTACAT 936
PI353814_R             TAATAGCCTAATAAATAGTACTTTAAAAAAATAATATTACACATAATTGTTTCTTTACAT 931
PI614596_S             TAATAGCCTAATAAATAGTACTTTAAAAAAATAATATTACACATAATTGTTTCTTTACAT 1440
                       ************************************************************

MELO3C022157Ref        ATTCCTAGAATCTCTGCTTGTTCTAAAAAGTCTCATGGAATATATAGAAAGGAGCCCGAC 996
PI353814_R             ATTCCTAGAATCTCTGCTTGTTCTAAAAAGTCTCATGGAATATATAGAAAGGAGCCCGAC 991
PI614596_S             ATTCCTAGAATCTCTGCTTGTTCTAAAAAGTCTCATGGAATATATAGAAAGGAGCCCGAC 1500
                       ************************************************************

MELO3C022157Ref        TAATTGAAGAATTAGATACACAAACTTTTTTAGAATTAGGAAAATAACTCTCTTCATTGA 1056
PI353814_R             TAATTGAAGAATTAGATACACAAACTTTTTTAGAATTAGGAAAATAACTCTCTTCATTGA 1051
PI614596_S             TAATTGAAGAATTAGATACACAAACTTTTTTAGAATTAGGAAAATAACTCTCTTCATTGA 1560
                       ************************************************************

MELO3C022157Ref        TGGTGTGGATTATATAGTGGGGATTCTGTTAATAGTCGTTCATTGTATACTATAGGAAGA 1116
PI353814_R             TGGTGTGGATTATATAGTGGGGATTCTGTTAATAGTCGTTCATTGTATACTATAGGAAGA 1111
PI614596_S             TGGTGTGGATTATATAGTGGGGATTCTGTTAATAGTCGTTCATTGTATACTATAGGAAGA 1620
                       ************************************************************

MELO3C022157Ref        AAGAAAGCGAATCCAAGCACACCATAAATTTTTTTTTGACATACTACTCTTTGGCCATGC 1176
PI353814_R             AAGAAAGCGAATCCAAGCACACCATAAATTTTAGTTTGACATACTACTCTTTGGCCATGC 1171
PI614596_S             AAGAAAGCGAATCCAAGCACACCATAAATTTTTTTTTGACATACTACTCTTTGGCCATGC 1680
                       ********************************  **************************

MELO3C022157Ref        AGCCCTGAAGTGGACAGTATCAACCGAATCACCAATCAAATATTTGATAAGTTGCGAAGA 1236
PI353814_R             AGCCCTGAAGTGGACAGTATCAACCGAATCACCAATCAAATATTTGATAAGTTGCGAAGA 1231
PI614596_S             AGCCCTGAAGTGGACAGTATCAACCGAATCACCAATCAAATATTTGATAAGTTGCGAAGA 1740
                       ************************************************************

MELO3C022157Ref        CCTATGTTAATAGGCCCTAATCAATTGAATTACTTGGTTGATATGCGAAGTAAGCTAAGG 1296
PI353814_R             CCTATGTTAATAGGCCCTAATCAATTGAATTACTTGGTTGATATGCGAAGTAAGCTAAGG 1291
PI614596_S             CCTATGTTAATAGGCCCTAATCAATTGAATTACTTGGTTGATATGCGAAGTAAGCTAAGG 1800
```

```
                     ************************************************************
MELO3C022157Ref      GATATCAATAACCTACTTGACTTTGAATCAGATGAAGTACGATTTATAGGAATAGTTGGA 1356
PI353814_R           GATATCAATAACCTACTTGACTTTGAATCAGATGAAGTACGATTTATAGGAATAGTTGGA 1351
PI614596_S           GATATCAATAACCTACTTGACTTTGAATCAGATGAAGTACGATTTATAGGAATAGTTGGA 1860
                     ************************************************************

MELO3C022157Ref      ATGGGTGGTATTGGTAAAACAACTATTGCAAAAGTTTTACACGACAGTATTGCATTTACA 1416
PI353814_R           ATGAGTGGTATTGGTAAAACAACTATTGCAAAAGTTTTACACGACAGTATTGCATTTACA 1411
PI614596_S           ATGGGTGGTATTGGTAAAACAACTATTGCAAAAGTTTTACACGACAGTATTGCATTTACA 1920
                     *** ********************************************************

MELO3C022157Ref      TTATTTGGTGAAAATTCTTGCTTTGTCACTATGTCTGGGCGTGATATCGTCACGGTCCAA 1476
PI353814_R           TTATTTGGTGAAAATTCTTGCTTTGTCACTATGTCTGGGCGTGATATCGTCACGGTCCAA 1471
PI614596_S           TTATTTGGTGAAAATTCTTGCTTTGTCACTATGTCTGGGCGTGATATCGTCACGGTCCAA 1980
                     ************************************************************
                                                 SNP at 2035th on susceptible parent
MELO3C022157Ref      TGTCTACTACTCTCTCGACTTCTTGGAACTAGGGAGAATATTAACATTTTAGAAAAGAAT 1536
PI353814_R           TGTCTACTACTCTCTCGACTTCTTGGAACTAGGGAGAATATTAACATTTTAGAAAAGAAT 1531
PI614596_S           TGTCTACTACTCTCTCGACTTCTTGGAACTAGGGAGAATATTAACATTTTAGAATAGAAT 2040
                     ****************************************************** *****

MELO3C022157Ref      GAAGGAGCAAACATGATTAAAGATTGTTTGAGTAGGAGAAAGGTTTTGATTATTTTTGAT 1596
PI353814_R           GAAGGAGCAAACATGATTAAAGATTGTTTGAGTAGGAGAAAGGTTTTGATTATTTTTGAT 1596
PI614596_S           GAAGGAGCAAACATGATTAAAGATTGTTTGAGTAGGAGAAAGGTTTTGATTATTTTTGAT 2100
                     ************************************************************

MELO3C022157Ref      GGGGTGAATGATAGAGAGGAATTAGGATACATAGCCGGAAGTTTTGATTGGTTTGGTCGA 1656
PI353814_R           GGGGTGAATGATAGAGAGGAATTAGGATACATAGCCGGAAGTTTTGATTGGTTTGGTCGA 1651
PI614596_S           GGGGTGAATGATAGAGAGGAATTAGGATACATAGCCGGAAGTTTTGATTGGTTTGGTCGA 2160
                     ************************************************************

MELO3C022157Ref      GGAAGTCGAGTCATCATTACCACTAGAAATAAAAATGTTTTTTCTCACCCCAATCATGAA 1716
PI353814_R           GGAAGTCGAGTCATCATTACCACTAGAAATAAAAATGTTTTTTCTCACCCCAATCATGAA 1711
PI614596_S           GGAAGTCGAGTCATCATTACCACTAGAAATAAAAATGTTTTTTCTCACCCCAATCATGAA 2220
                     ************************************************************

MELO3C022157Ref      CAAGTTCAACTCTACAATGTGAAACCACTTGATTACAACACTTCATTCTCACTTTTTTGG 1776
PI353814_R           CAAGTTCAACTCTACAATGTGAAACCACTTGATTACAACACTTCATTCTCACTTTTTTGG 1771
PI614596_S           CAAGTTCAACTCTACAATGTGAAACCACTTGATTACAACACTTCATTCTCACTTTTTTGG 2280
                     ************************************************************

MELO3C022157Ref      AAGCATGCATTTGATCAACAAAGTGGGGGTCCAAGTGAACAACAATTCATACAACTTAGT 1836
PI353814_R           AAGCATGCATTTGATCAACAAAGTGGGGGTCCAAGTGAACAACAATTCATACAACTTAGT 1831
PI614596_S           AAGCATGCATTTGATCAACAAAGTGGGGGTCCAAGTGAACAACAATTCATACAACTTAGT 2340
                     ************************************************************

MELO3C022157Ref      CAGAATATAGTGGAAAAGGTCGAAGGAAATCCACAAGCATTGGAACAAATTGGATCATTT 1896
PI353814_R           CAGAATATAGTGGAAAAGGTCGAAGGAAATCCACAAGCATTGGAACAAATTGGATCATTT 1891
PI614596_S           CAGAATATAGTGGAAAAGGTCGAAGGAAATCCACAAGCATTGGAACAAATTGGATCATTT 2400
                     ************************************************************

MELO3C022157Ref      TTGCGTGGTAAAGATATTAATGTATGGAAAGAAGAATTGAAGAGCCTTGTTTTAGTTGAT 1956
PI353814_R           TTGCGTGGTAAAGATATTAATGTATGGAAAGAAGAATTGAAGAGCCTTGTTTTAGTTGAT 1951
PI614596_S           TTGCGTGGTAAAGATATTAATGTATGGAAAGAAGAATTGAAGAGCCTTGTTTTAGTTGAT 2460
                     ************************************************************

MELO3C022157Ref      AATGAACGTCTCTTCAAAATATTAAAGATAAGTTTTGATCAATTAGGGACAAAAGGCCAA 2016
PI353814_R           AATGAACGTCTCTTCAAAATATTAAAGATAAGTTTTGATCAATTAGGGACAAAAGGCCAA 2011
PI614596_S           AATGAACGTCTCTTCAAAATAT----GATAAGTTTTGATCAATTAGGGACAAAAGGCCAA 2516
                     **********************    **********************************

MELO3C022157Ref      CAAGCTTTTCTTGATTTGGCATGTTTCTTCAATGGAAAAAGTACAGGCAAAATTATTGAA 2076
PI353814_R           CAAGCTTTTCTTGATTTGGCATGTTTCTTCAATGGAAAAAGTACAGGCAAAATTATTGAA 2071
PI614596_S           CAAGCTTTTCTTGATTTGGCATGTTTCTTCAATGGAAAAAGTACAGGCAAAATTATTGAA 2576
                     ************************************************************

MELO3C022157Ref      ATACTTGCGAGTTTAGAATACAATTCCCCCAGCGAAGTACTAAAGTTGTTGTGTGATAGA 2136
PI353814_R           ATACTTGCGAGTTTAGAATACAATTCCCCCAGCGAAGTACTAAAGTTGTTGTGTGATAGA 2131
PI614596_S           ATACTTGCGAGTTTAGAATACAATTCCCCCAGCGAAGTACTAAAGTTGTTGTGTGATAGA 2636
                     ************************************************************

MELO3C022157Ref      TATCTTATTGAAATTAGAGATGGAGACACAGTATGTATGCCCAATTTGATACAAGAAATG 2196
PI353814_R           TATCTTATTGAAATTAGAGATGGAGACACAGTATGTATGCCCAATTTGATACAAGAAATG 2191
PI614596_S           TATCTTATTGAAATTAGAGATGGAGACACAGTATGTATGCCCAATTTGATACAAGAAATG 2696
                     ************************************************************

MELO3C022157Ref      GGTCGAGAAATAGAACGAAAAAAACGTCAAAGAAGCAGGATTTGGCTTAGAAGAGATGCT 2256
PI353814_R           GGTCGAGAAATAGAACGAAAAAAACGTCAAAGAAGCAGGATTTGGCTTAGAAGAGATGCT 2251
PI614596_S           GGTCGAGAAATAGAACGAAAAAAACGTCAAAGAAGCAGGATTTGGCTTAGAAGAGATGCT 2756
                     ************************************************************

MELO3C022157Ref      TTCGACATATTTGATGAACAACATGTAAGTCTATATATGTTACATTATATTATTGTACAT 2316
PI353814_R           TTCGACATATTTGATGAACAACATGTAAGTCTATATATGTTACATTATATTATTGTACAT 2311
PI614596_S           TTCGACATATTTGATGAACAACATGTAAGTCTATATATGTTACATTATATTATTGTACAT 2816
                     ************************************************************

MELO3C022157Ref      TATTTTCCATGTTATTTAATTATATTAACTGATTTACTTTGGCAGGGAGTAAAAGACATA 2376
PI353814_R           TATTTTCCATGTTATTTAATTATATTAACTGATTTACTTTGGCAGGGAGTAAAAGACATA 2371
PI614596_S           TATTTTCCATGTTATTTAATTATATTAACTGATTTACTTTGGCAGGGAGTAAAAGACATA 2876
```

```
                     ************************************************************
MELO3C022157Ref      AAAGGTGTTGTGTTGGACAAGAGAGACACAGAACCAAACTTAAAGTTGAAGGCTAAACAA 2436
PI353814_R           AAAGGTGTTGTGTTGGACAAGAGAGACACAGAACCAAACTTAAAGTTGAAGGCTAAACAA 2431
PI614596_S           AAAGGTGTTGTGTTGGACAAGAGAGACACAGAACCAAACTTAAAGTTGAAGGCTAAACAA 2936
                     ************************************************************
MELO3C022157Ref      TTACAAGATATGAGCCGTTTAAAAATATTAGAGATTGACAATGTGCAGCTGAGTCCAAGA 2496
PI353814_R           TTACAAGATATGAGCCGTTTAAAAATATTAGAGATTGACAATGTGCAGCTGAGTCCAAGA 2491
PI614596_S           TTACAAGATATGAGCCGTTTAAGAATATTAGAGATTGACAATGTGCAGCTGAGTCCAAGA 2996
                     ********************** *************************************
MELO3C022157Ref      AATCAAAATGATCTCTCAAATCAGCTTCGATTGCTCCACTGGGATGGCTTTCCTTCAGAC 2556
PI353814_R           AATCAAAATGATCTCTCAAATCAGCTTCGATTGCTCCACTGGGATGGCTTTCCTTCAGAC 2551
PI614596_S           AATCAAAATGATCTCTCAAATCAGCTTCGATTGCTCCACTGGGATGGCTTTCCTTCAGAC 3056
                     ************************************************************
MELO3C022157Ref      ACTTTGCCACTAAATTTCGAAGCACCATATTTATTTGAACTTCTCTTGCCTAATGCACAA 2616
PI353814_R           ACTTTGCCACTAAATTTCGAAGCACCATATTTATTTGAACTTCTCTTGCCTAATGCACAA 2611
PI614596_S           ACTTTGCCACTAAATTTCGAAGCACCATATTTATTTGAACTTCTCTTGCCTAATGCACAA 3116
                     ************************************************************
MELO3C022157Ref      ACCACTCATCTTTGGAAAGAACTAAAGGTTAGACATATATTTGCAAATACTTTAACCTGC 2676
PI353814_R           ACCACTCATCTTTGGAAAGAACTAAAGGTTAGACATATATTTGCAAATACTTTAACCTGC 2671
PI614596_S           ACCACTCATCTTTGGAAAGAACTAAAGGTTAGACATATATTTGCAAATACTTTAACCTGC 3176
                     ************************************************************
MELO3C022157Ref      GCTATATATATATATATGTTGTGATTGTGCTATTTGCAATAATTTTTCAAATTTTCTTTC 2736
PI353814_R           GCTATATATATATATATGTTGTGATTGTGCTATTTGCAATAATTTTTCAAATTTTCTTTC 2731
PI614596_S           GCTATATATATATATATGTTGTGATTGTGCTATTTGCAATAATTTTTCAAATTTTCTTTC 3236
                     ************************************************************
MELO3C022157Ref      ATAACATATATAAATACATGTTTTGTTATTTGGTAACAGGGATTTAAGAAATTAAAGGTA 2996
PI353814_R           ATAACATATATAAATACATGTTTTGTTATTTGGTAACAGGGATTTAAGAAATTAAAGGTA 2991
PI614596_S           ATAACATATATAAATACATGTTTTGTTATTTGGTAACAGGGATTTAAGAAATTAAAGGTA 3296
                     ************************************************************
MELO3C022157Ref      ATCGATGTTAGCAATTCCCAAACTTTGGTGGAGACACCGAATTTGAGTGCTGTTCCAAAT 2856
PI353814_R           ATCGATGTTAGCAATTCCCAAACTTTGGTGGAGACACCGAATTTGAGTGCTGTTCCAAAT 2851
PI614596_S           ATCGATGTTAGCAATTCCCAAACTTTGGTGGAGACACCGAATTTGAGTGCTGTTCCAAAT 3356
                     ************************************************************
MELO3C022157Ref      CTAGAAAGATTGATTCTATGTAATTGTACAAGATTGAAGAAAATTGATAATTCAATTACA 2916
PI353814_R           CTAGAAAGATTGATTCTATGTAATTGTACAAGATTGAAGAAAATTGATAATTCAATTACA 2911
PI614596_S           CTAGAAAGATTGATTCTATGTAATTGTACAAGATTGAAGAATATTGATAATTCAATTACA 3416
                     ***************************************** ******************
MELO3C022157Ref      AAATTGAGACTTCTAGTTTTAGTAGACCTCACAGGCTGTGTTCGCCTCGAAACATCCGAG 2976
PI353814_R           AAATTGAGACTTCTAGTTTTAGTAGACCTCACAGGCTGTGTTCGCCTCGAAACATCCGAG 2971
PI614596_S           AAATTGAGACTTCTAGTTTTAGTAGACCTCACAGGCTGTGTTCGCCTCGAAACATCCGAG 3476
                     ************************************************************
MELO3C022157Ref      TGCATCGATATCTTGAAGAGTCGCCCAACAGTAGAACTTCGTGGCTTAGTTCTACAGTGT 3036
PI353814_R           TGCATCGATATCTTGAAGAGTCGCCCAACAGTAGAACTTCGTGGCTTAGTTCTACAGTGT 3031
PI614596_S           TGCATCGATATCTTGAAGAGTCGCCCAACAGTAGAACTTCGTGGCTTAGTTCTACAGTGT 3536
                     ************************************************************
MELO3C022157Ref      CGCCAATCATTGAAAGGTATTTGTTATATTCGTAAATTTTTTCACTTCTTTTTTAAAATT 3096
PI353814_R           CGCCAATCATTGAAAGGTATTTGTTATATTCGTAAATTTTTTCACTTCTTTTTTAAAATT 3091
PI614596_S           CGCCAATCATTGAAAGGTATTTGTTATATTCGTAAATTTTTTCACTTCTTTTTTAAAATT 3596
                     ************************************************************
MELO3C022157Ref      CAGGATCATTTTGTGTAA 3114
PI353814_R           CAGGATCATTTTGTGTAA 3109
PI614596_S           CAGGATCATTTTGTGTAA 3614
                     ******************
```

(c)InterProScan (protein)

Reference (MELO3C022157)

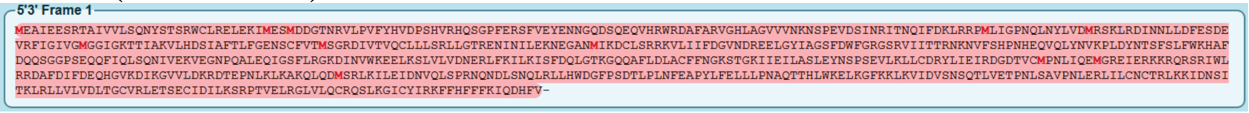

Resistant Parent

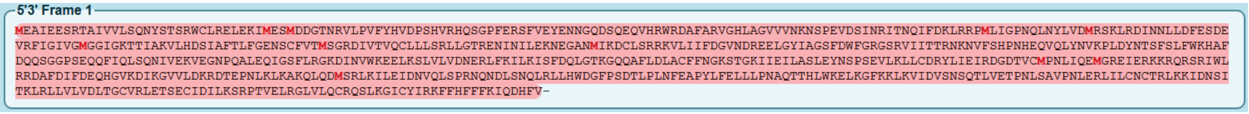

Susceptible Parent

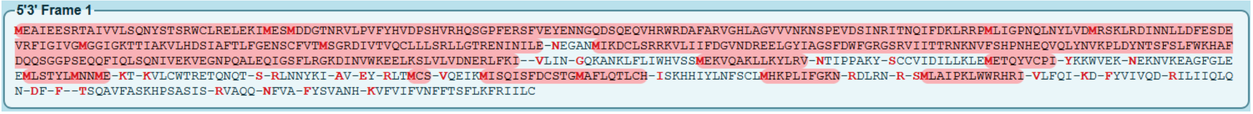

Figure S6: Sequences and alignments of cloned and sequenced TIR-NBS-LRR gene MELO3C022157 from resistant (PI353814) and susceptible (PI614596) melon accessions. (a) Genomic sequences of reference, resistant and susceptible accessions. (b) Their alignment with reference sequences retrieved from the Cucurbit Genomics database (http://cucurbitgenomics.org) considering DHL92 as the reference genome. In the sequence alignment, asterisks (*) indicate sequence similarity and absence of asterisks indicates sequence dissimilarity; em-dash (—) and green color designate insertion/deletion of nucleotides. In the gDNA sequences, grey highlights illustrate the position of exons, yellow highlights indicate InDel primer (MB157-2-F and MB157-2-R) sequences and light blue indicates the primer name and InDel position. (c) Translated protein sequences of reference gene, resistant and susceptible parent sequences determined using the "Translate Tool-ExPASy" (https://web.expasy.org/translate/) web-based database and showing amino acid alterations and truncated protein in the susceptible parent.
